# The effect of different resistance and assistance loads on 30-m sprint kinematics

**DOI:** 10.1371/journal.pone.0298517

**Published:** 2024-03-22

**Authors:** Fuzong Chang, Dexing Qian, Shouxin Zong, Yanfei Shen

**Affiliations:** School of Sports Engineering, Beijing Sport University, Beijing, China; Jerzy Kukuczka Academy of Physical Education In Katowice: Akademia Wychowania Fizycznego imienia Jerzego Kukuczki w Katowicach, POLAND

## Abstract

Resisted sprint and assisted sprint are the two main types of training methods used by athletes in sprint training, so optimizing resisted sprint training and assisted sprint training process is beneficial for improving athletes’ sprint performance. Kinematics is the most intuitive parameter that reflects the quality of training during running process, and it is particularly important to analyze the gait of athletes during resisted and assisted sprint process. Therefore, this paper investigates the effects of resisted and assisted sprint on the sprint kinematics of sprinters in the first 30 meters to demonstrate the targeted effects of resisted and assisted sprint training. The experimental results show that compared to the unloaded running, male collegiate sprinters increase their total step count, decrease their step length, increase their step time, increase their contact time, whereas have almost no change in the flight time when performing the 30-m resisted sprint. Male collegiate sprinters decrease their total step count, increase their step length, increase their step time, decrease their contact time and increase their flight time, when performing the 30-m assisted sprint. In addition, it is found that resisted sprint training is beneficial for improving the athletes’ power and explosiveness during the acceleration phase, thereby improving acceleration ability. However, prolonged and frequent resisted sprint training may reduce the step length and step frequency of athletes. Assisted sprint training is beneficial for shortening the contact time of athletes, improving their step length and flight time, and enabling them to overspeed, thereby increasing their maximum speed ability.

## Introduction

In various types of sports, the short distance sprint ability of athletes is a key factor in outperforming opponents to win a race, achieve great results or score goals [1, 2]. Resisted sprint and assisted sprint are the two main types of training methods used to improve sprint ability [3, 4], which provide appropriate interventions and loads for different aspects of the athlete muscular development [5]. As for resisted sprint training, it can replicate the movement patterns of sprinting, while improving muscle strength, peak force and muscle nerve recruitment through horizontal resistance stimulation [6, 7]. As for assisted sprint training improves the athlete’s speed and step length by providing overdrive conditions through assistance, allowing the muscles to adapt to faster speeds and longer step lengths, thereby improving the athlete’s maximum speed ability and developing good running posture [8]. Resisted sprint training is currently implemented in a number of ways, such as athletes running in equipment like weighted vests and weighted sandbags [9], having athletes pull types or resistance umbrellas for sprint training process [10], using elastic bands for others to pull backwards to provide resistance, and using pulley systems [4]. There are also a number of different ways to achieve assistance training, including running downwind under certain weather conditions and downhill running on certain surfaces [11], as well as assistance running, which is an important form of training where the athlete is pulled forward by another person to provide forward assistance [4].

Analysis of the movements of athletes with different performance levels showed that elite athletes have a lower torso angle and are more upright than average athletes during sprint process. Wearing a resistance parachute and weighted vest can cause the athlete to lean forward excessively during the sprint process, affecting the basic sprint movement pattern. However, sleds and resistance training equipment can avoid the effect on the athlete’s trunk angle effectively by placing load on the waist and below [10]. Resistance training aims to increase athletes’ running speed by overloading the muscles, which leads to greater neural activation and faster recruitment of muscle fibers. During the resistance training process with load, it is expected to potentially decrease the athlete’s running speed, step length, and step frequency. This reduction in running speed and step parameters is attributed to the added resistance that the muscles have to overcome, resulting in a temporary decrease in performance during the training session. However, over time, as the muscles adapt and become stronger, the athlete’s running speed and step parameters are expected to improve, leading to enhanced performance. The load applied to the athletes reduces the distance that they can move forward during the flight phase, thereby reducing the athlete’s step length. In order to maintain the same movement pattern and range of motion during the contact phase, the athletes need to generate more horizontal force to counter the load, thereby increasing the contact time. The result of longer ground contact time and almost constant flight time is a reduction in step frequency [12].

Research has shown that the overall trend of kinematic parameters during assisted sprint is consistent with normal sprint during the ground contact phase. Assisted sprint does not alter the technical structure of sprint movements and exhibits similarities to normal sprint techniques. It creates neural adaptations in the athlete through over-speed sprint, making it an effective specialized training method for sprint [8].

Currently, most research on resisted and assisted sprint training involves implementing training interventions for athletes using tools such as resistance sleds, elastic bands, resistance parachutes, and weighted vests. These studies primarily focus on assessing changes in athletes’ sprint times and performance, with the aim of evaluating post-activation performance enhancement or longitudinal training effects [13–17]. The majority of these investigations concentrate solely on changes over specific distances, without providing any insight into the kinematics and biomechanics of the study. Some studies in the field of kinematics have found that the time differences between regular sprints and resisted sprints are primarily attributed to a decrease in step length and step frequency under resisted conditions [18]. In contrast, assisted sprint requires less time to complete, with longer step lengths, extended flight times, and shorter contact times under assistance conditions [19–21]. However, few studies explored both the effects of resistance conditions and assistance conditions with different loads on sprint kinematics. Assessing the acute effects of varied resistance and assistance loads on sprint kinematics during sprint could be instrumental in devising tailored training programs aimed at enhancing sprint technique and performance. Therefore, the aim of this study is to investigate the effects of different resistance and assistance loads on the sprint kinematics of male collegiate sprinters during the 30-m sprint. It is hypothesized that resistance and assistance conditions have different effects on the total time, step length, step time, contact time, and flight time in the 30-m sprint for male collegiate sprinters.

## Materials and methods

Traditional training methods that load athletes by dragging weighted sleds, wearing weighted vests, running uphill and downhill, and elastic band-assisted running are difficult to quantify and replicate. In the study of sprint kinematics changes, the load must be precisely controlled to ensure the feasibility of replicating the study [22]. With the development of sports technology, digital training and testing systems, such as the 1080Sprint^™^ and DynaSpeed^™^, are now used for sprint training [23–25]. They can provide constant resistance and assistance throughout the entire sprint training by using motors to provide resistance or assistance. Jueying is a digital training and testing system developed independently by Beijing Sport University, also belongs to this category and allows athletes to perform resisted and assisted sprint training with precisely controlled loads. The equipment works by attaching one end of a cord to the roller of the equipment and the other end to a belt worn by the athlete. The roller is driven by a servo motor, thereby providing precise and constant resistance. A high-speed camera (Z CAM E2-M4, resolution 1080p, frame rate 240 fps) recorded the sprint videos. Sprint kinematics were captured by analyzing the video frame by frame using Kinovea (v0.9.5) video analysis software, which is a widely used tool for its precise and efficient motion analysis capabilities.

### Ethical considerations and approvals

This study has obtained ethical approval with the approval number 2022235H, issued by Sports Science Experiment Ethics Committee of Beijing Sport University.

Participants were provided with detailed information about the study and were asked to give informed consent before participating in the study.

The principles of confidentiality and privacy protection are strictly observed.

Ongoing monitoring and review were be conducted to address any ethical issues that may arise during the course of the study.

### Experimental standards

In the case of resisted sprint, the selection of loads—3 kg, 7 kg, and 14 kg—derives from a meticulous consideration of established principles. It has been proposed that 10% BW(Body Weight) is the optimal resistance load for resisted sprint training, as this load has been shown not to significantly affect sprint mechanics [7, 26]. This guideline is consistent with the utilization of a 7 kg load in our study. Biomechanical research in resisted sprint suggests that employing a higher resistance load, such as 20% BW, can yield more substantial biomechanical data [27]. providing justification for the inclusion of a 14 kg load in our study. Recognizing that even loads equivalent to 10% BW are classified as high-intensity exercise, the incorporation of a 3 kg load enables a comprehensive exploration of potential changes and facilitates a nuanced understanding of the effects of varying resistance loads on athletes.

As for assisted sprint, we used loads of 7 kg, 9 kg, and 11 kg, different from the loads used in resisted sprint. Previous studies on assisted sprint have used assistance loads of 3 kg, 4 kg, 5 kg [21] as well as 8 kg [22]. Since the athlete did not reach maximum speed during the 30 m sprint, small assistance loads, such as 3 kg, may not have an observable effect on the kinematic data. On the other hand, large assistance loads, such as 14 kg, could seriously disrupt the athlete’s technical movement. To systematically explore the impact of progressively increasing assistance, we selected larger assistance loads based on existing research. The loads of 7 kg, 9 kg, and 11 kg establish a gradual progression, facilitating a comprehensive understanding of the changes in kinematics under progressively increasing assistance conditions.

In each experiment, the Jueying equipment and high-speed camera are placed at the same position and angle on the experimental site to ensure the minimization of errors in data acquisition. The subjects selected a 20-minute warm-up before the experiment process, followed by a 10-minute sprint warm-up led by the staff. The equipment operator repairs and calibrates the equipment before starting the experiment process. The Jueying equipment provided resistance load at 3 kg, 7 kg and 14 kg, and provided assistance load at 7 kg, 9 kg and 11 kg, and obtained segmented and total time data. Kinovea software automatically captured the reflective marker points with the help of manual calibration to obtain the subject’s segmented time, total time, step length, step time, contact time and flight time for each step. Step length refers to the distance between the two toe in contact at each step, contact time refers to the time between the same toe coming into contact and the time the subject leaves the ground, flight time refers to the time between the subject’s toe leaving the ground and the other toe touching the ground, and step time refers to the sum of contact time and flight time.

### Experimental process

University Institutional Review Board approval was obtained. Fourteen male collegiate sprinters (age 21±2 years, weight 69.6±5.3 kg, height 179.1±4.1cm) with extensive training experience participated in this experiment. All subjects were national-level 2 athletes (with the times between 10.8 and 11.74 seconds) in the 100-meter event. They had previous experience in 30-meter sprints as well as resistance and assistance training. Prior to the testing process, the subjects were informed about the procedures and potential risks and provided their informed consent. They wore consistent gym clothes and spiked shoes for each session. The subjects were instructed to maintain good physical condition and avoid maximum load training within 48 hours before the testing process. The testing sessions for each subject had an interval of more than 48 hours and were conducted at approximately the same time of day (±0.5 hour) in the same testing environment to minimize the confounding effects of circadian rhythm changes and environmental factors [28]. The study was conducted from January 3 to January 20, 2023.

Before each experiment, the subjects were briefed on the specific procedures, load requirements, movement essentials, and technical specifications. It was ensured that the apparatus and equipment were properly used and free of any safety hazards. To reduce the risk of injury to the posterior thigh muscles, knee joints, and ankle joints during resistance running, the subjects performed a 20-minute self-selected warm-up followed by a 10-minute sprint warm-up consisting of: jogging, butt kicks, high knees with sprint arm action, walking lunges, A skips, leg swings, and dynamic stretches.

The experiment sessions were conducted in a random order for all subjects. The first experiment involved an unloaded 30-meter sprint test, followed by a rest period of 6–8 minutes before starting the next experiment. To avoid the post-activation effect of resistance training [29], each resistance experiment used only one load on the subjects. The first experiment used a resistance load of 3 kg, the second experiment used a resistance load of 7 kg, and the third experiment used a resistance load of 14 kg. The assistance testing process, which was the fourth experiment, involved testing the subjects with assistance loads of 7 kg, 9 kg, and 11 kg on the same day, with a rest interval of 6–8 minutes between each test to prevent fatigue. For the assistance experiments, the Jueying equipment was connected to the sprint belt worn by the subjects using a hook and loop mechanism to provide assistance. The starting position for each test was marked on the field, with marker barrels placed at the start line and at positions 5, 10, 15, 20, 25, and 30 meters from the start line. In the resistance testing process, the Jueying equipment was positioned 10 meters behind the start line to provide resistance, and the participants sprinted in the opposite direction. In the assistance testing process, the Jueying equipment was placed 80 meters ahead of the start line to provide assistance, and the participants sprinted towards the equipment.

The high-speed camera was fixed on a tripod positioned perpendicular to the runway, approximately 15 meters away from a marked barrel at 25 meters. It was able to capture the entire 30-meter distance in its lens, and each test was conducted from the same position and angle. Reflective markers were applied to the hip, knee, ankle, and toe of the subjects on the side facing the high-speed camera. The camera was synchronized with the Jueying equipment and automatically recorded a video of the subject during each sprint.

Since the position of the high-speed camera was fixed on the tripod, a visual angle was created for each marker barrel, resulting in a distance error [30]. When the subject was sprinting within the view of the high-speed camera, if the hook and loop of the belt aligned with the projected line from the set marker barrel, it could be determined, based on the spatial geometry law, that the subject was precisely at the position of each segment at that moment.

**Fig 1:**
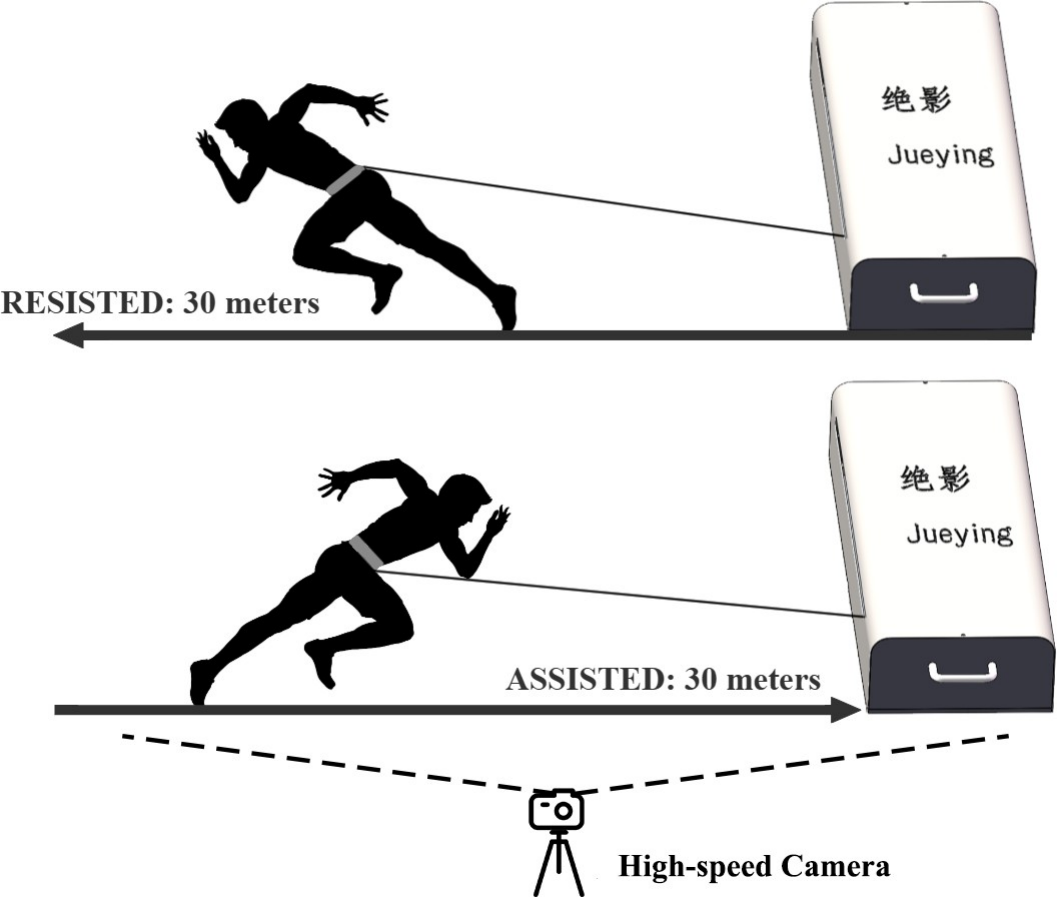
Jueying equipment for sprint experiments.

All subjects were instructed to start from a standing position with both feet behind the starting line and the toes of the front foot pressed against the starting line, as shown in Fig 1.

### Experimental data

Data processing is completed by using SPSS 26 (SPSS Inc., United States) software. In order to compare the effects of different loading conditions on sprint time and gait parameters, paired samples t-tests are performed and Cohen’s d values were calculated, which are the effect size based on the Hopkins Modified Cohen Scale. The magnitude of the effect of different loads on the data is evaluated by using Cohen’s d as an effect size. In this paper, Cohen’s d values less than 0.20 are considered very small, values in the range of 0.20–0.49 are considered small, values in the range of 0.50–0.79 are considered medium, and values greater than 0.80 are considered large.

### Experimental results

The mean values (mean ± SD) of the segmented time, total time, absolute variation, and relative variation of the sprint differed between loading conditions (Table 1). The sprint times for the 0–30 meters segment in the 3 kg, 7 kg, and 14 kg resistance tests were 12.13%, 22.68%, and 43.08% slower, respectively, compared to the no-load test. In contrast, the sprint times for the 0–30 meters segment in the 7 kg, 9 kg, and 11 kg assistance tests were 8.06%, 9.74%, and 10.71% faster, respectively, compared to the no-load test. The relative differences in segmented times were greatest in the 0–10 meters segment for the resistance conditions compared to the no-load condition, while the greatest relative differences were observed in the 20–30 meters segment for the assistance conditions compared to the no-load condition.

**Table 1:** Variation of segmentation time and total time under different load conditions.

	Unload	Resistance Load: 3kg	Resistance Load: 7kg	Resistance Load: 14kg	Assistance Load: 7kg	Assistance Load: 9kg	Assistance Load: 11kg
*Durationof*0 − 10*meters*(*s*)	1.65±0.02	1.99±0.02	2.23±0.03	2.45±0.04	1.51±0.02	1.50±0.02	1.49±0.02
*Absolutevariationtime*(*s*)		0.34±0.02	0.58±0.03	0.80±0.03	0.14±0.02	0.15±0.03	0.16±0.03
*Relativevariationrate*(%)		20.54±1.14	35.35±2.11	48.68±1.86	8.57±1.23	9.00±1.60	9.69±1.72
*Durationof*10 − 20*meters*(*s*)	1.28±0.04	1.36±0.04	1.45±0.05	1.70±0.07	1.19±0.04	1.16±0.04	1.15±0.04
*Absolutevariationtime*(*s*)		0.08±0.03	0.17±0.05	0.43±0.05	0.08±0.03	0.11±0.04	0.12±0.05
*Relativevariationrate*(%)		6.42±2.28	13.67±3.72	33.80±4.21	6.42±2.04	8.82±2.70	9.43±3.58
*Durationof*20 − 30*meters*(*s*)	1.20±0.03	1.28±0.04	1.38±0.05	1.69±0.07	1.09±0.05	1.06±0.04	1.04±0.04
*Absolutevariationtime*(*s*)		0.08±0.03	0.18±0.05	0.50±0.06	0.11±0.04	0.14±0.04	0.15±0.04
*Relativevariationrate*(%)		6.71±2.97	14.95±4.29	41.88±4.76	9.06±3.44	11.68±2.88	12.88±3.35
*Durationof*0 − 30*meters*(*s*)	4.12±0.09	4.72±0.08	5.06±0.12	5.90±0.17	3.79±0.10	3.72±0.10	3.68±0.08
*Absolutevariationtime*(*s*)		0.6±0.06	0.94±0.12	1.78±0.13	0.33±0.08	0.40±0.09	0.44±0.09
*Relativevariationrate*(%)		12.13±1.70	22.68±2.99	43.08±3.20	8.06±1.93	9.74±2.16	10.71±2.67

**Fig 2:**
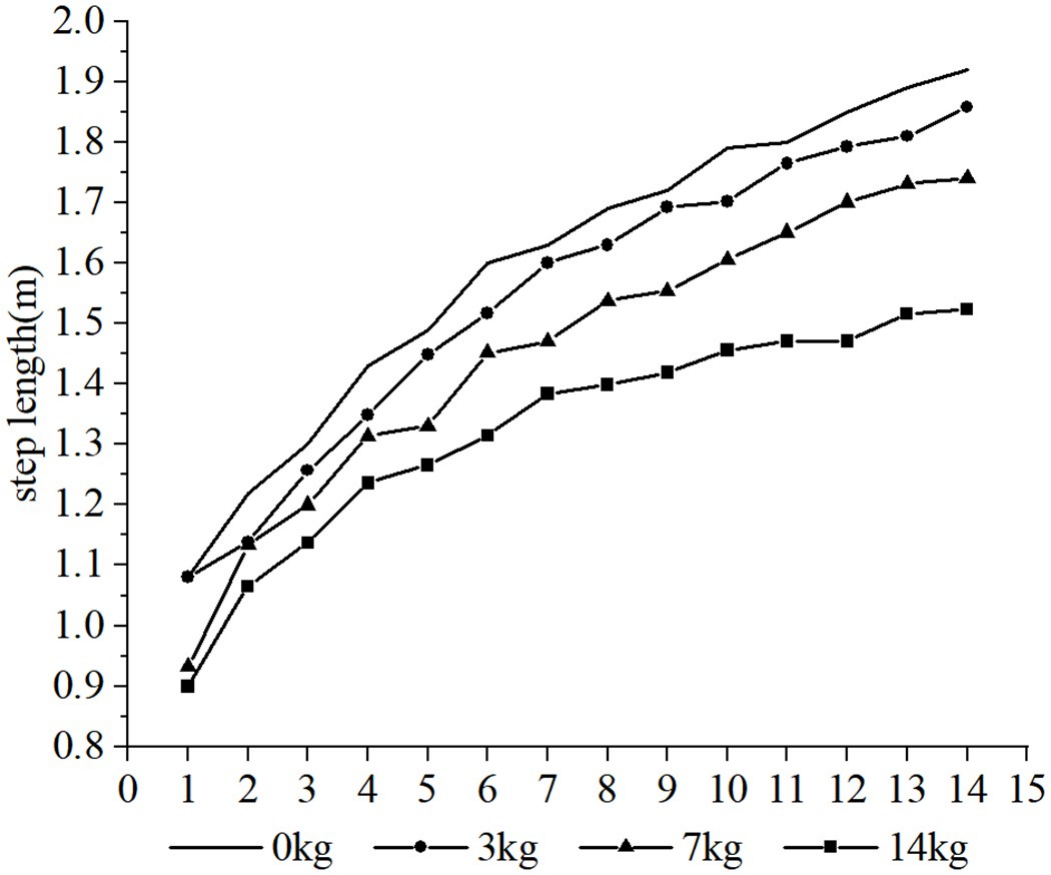
The effect of different resistance loads on step length.

**Fig 3:**
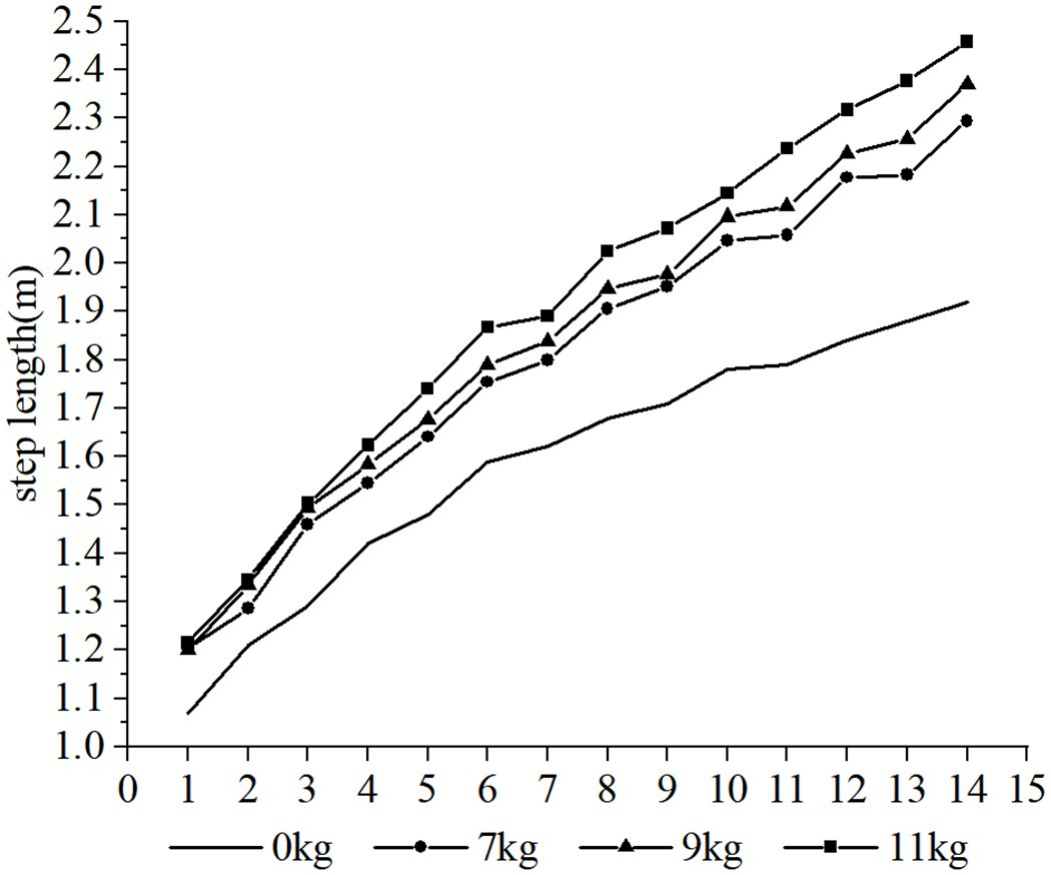
The effect of different assistance loads on step length.

**Fig 4:**
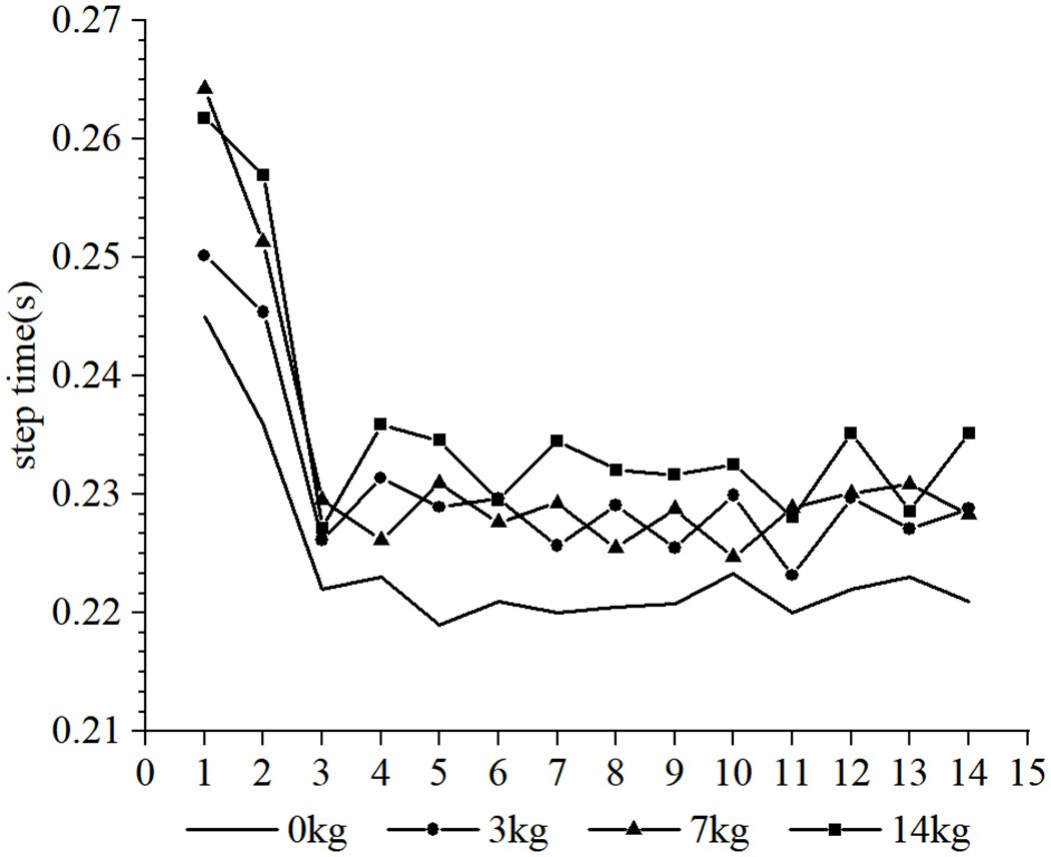
The effect of different resistance loads on step time.

**Fig 5:**
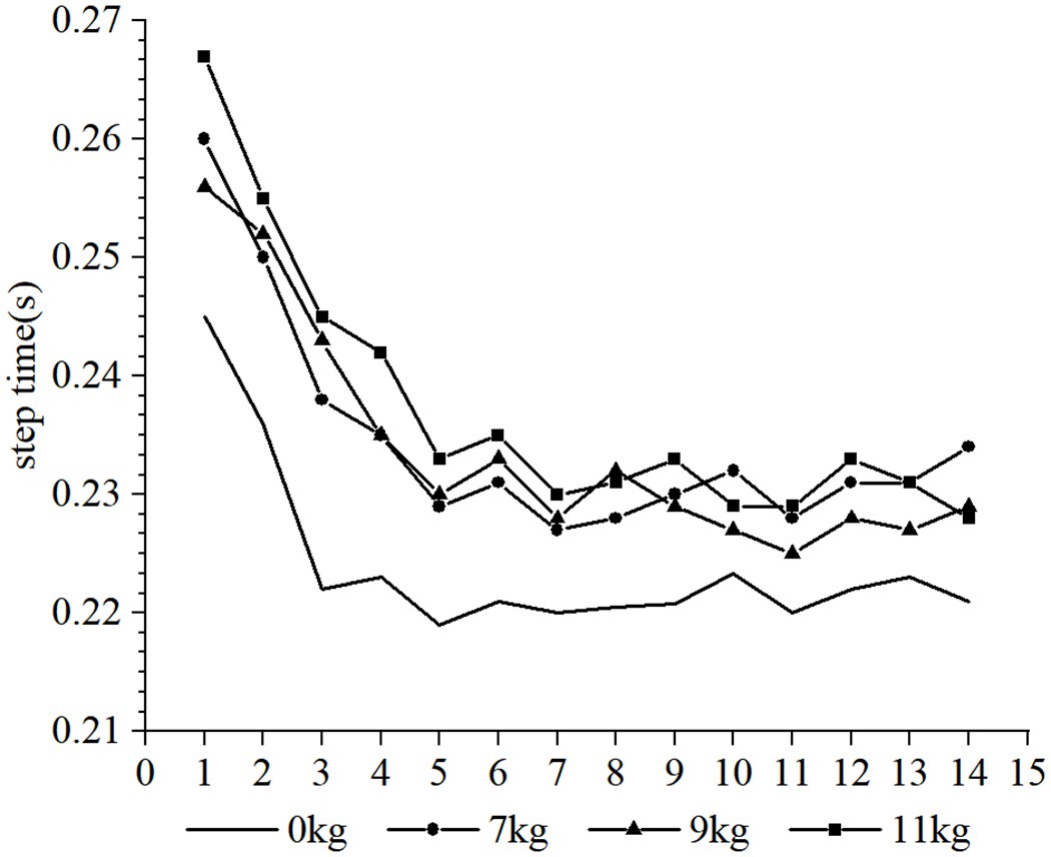
The effect of different assistance loads on step time.

**Fig 6:**
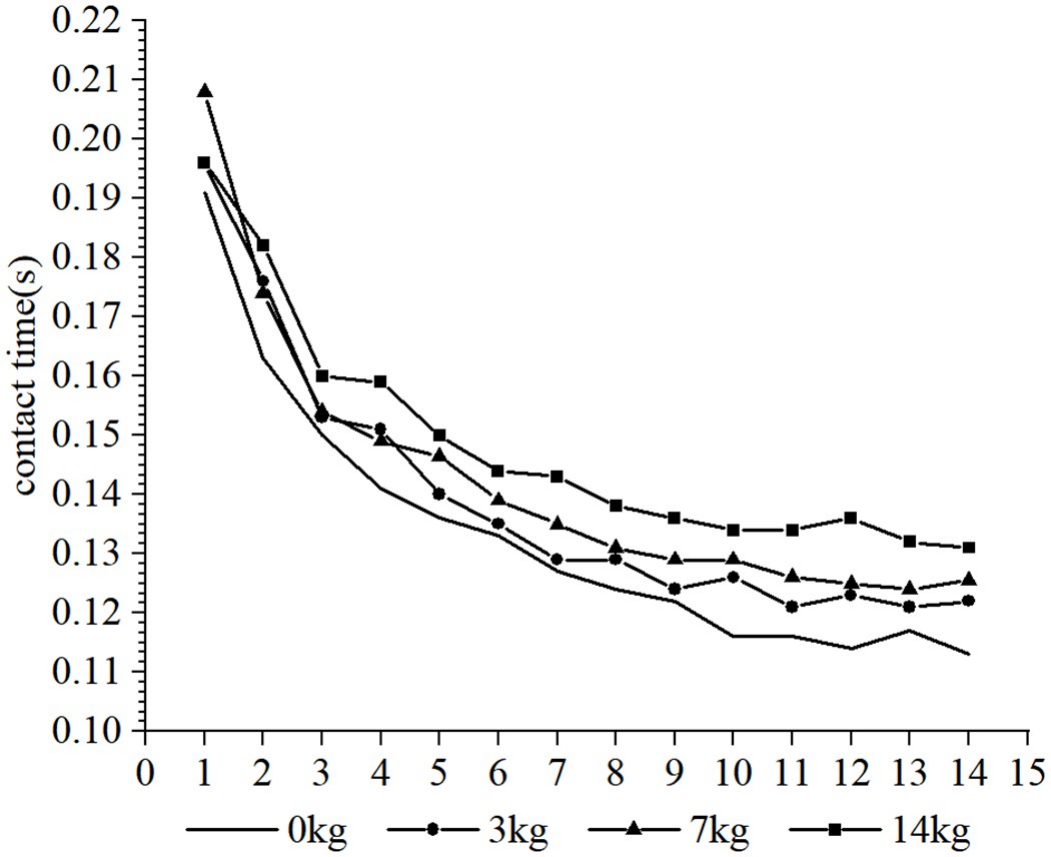
The effect of different resistance loads on contact time.

**Fig 7:**
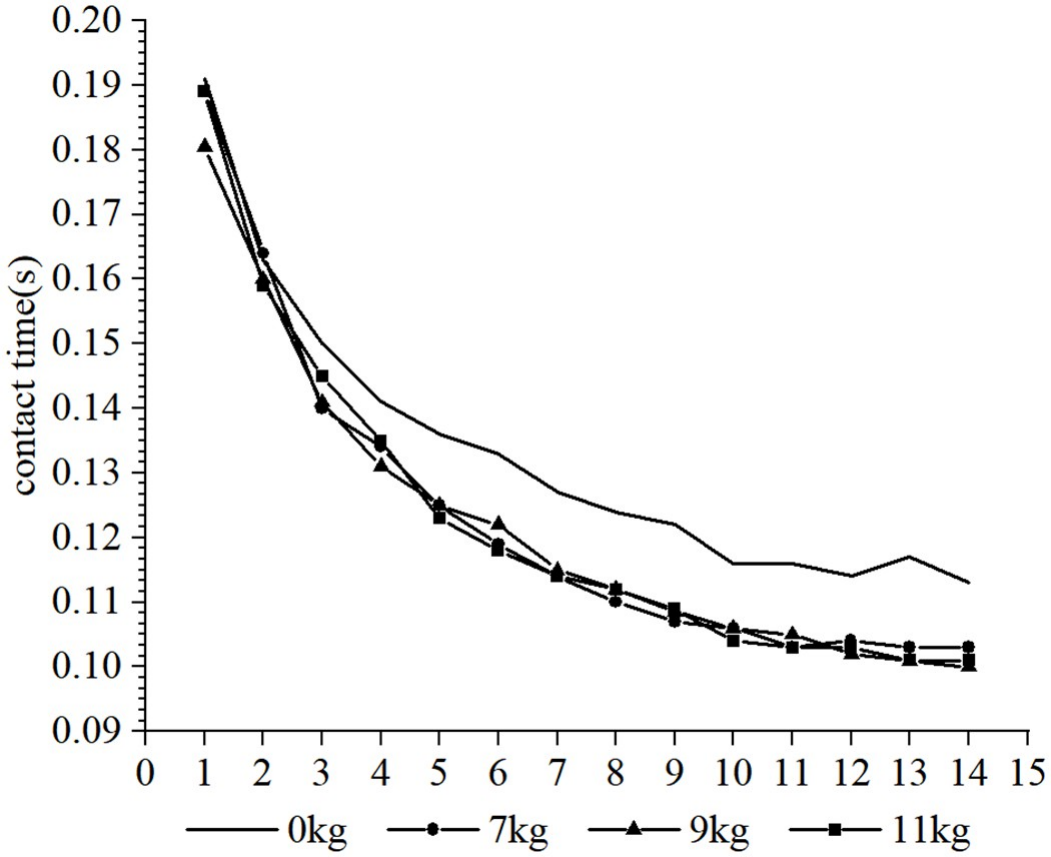
The effect of different assistance loads on contact time.

**Fig 8:**
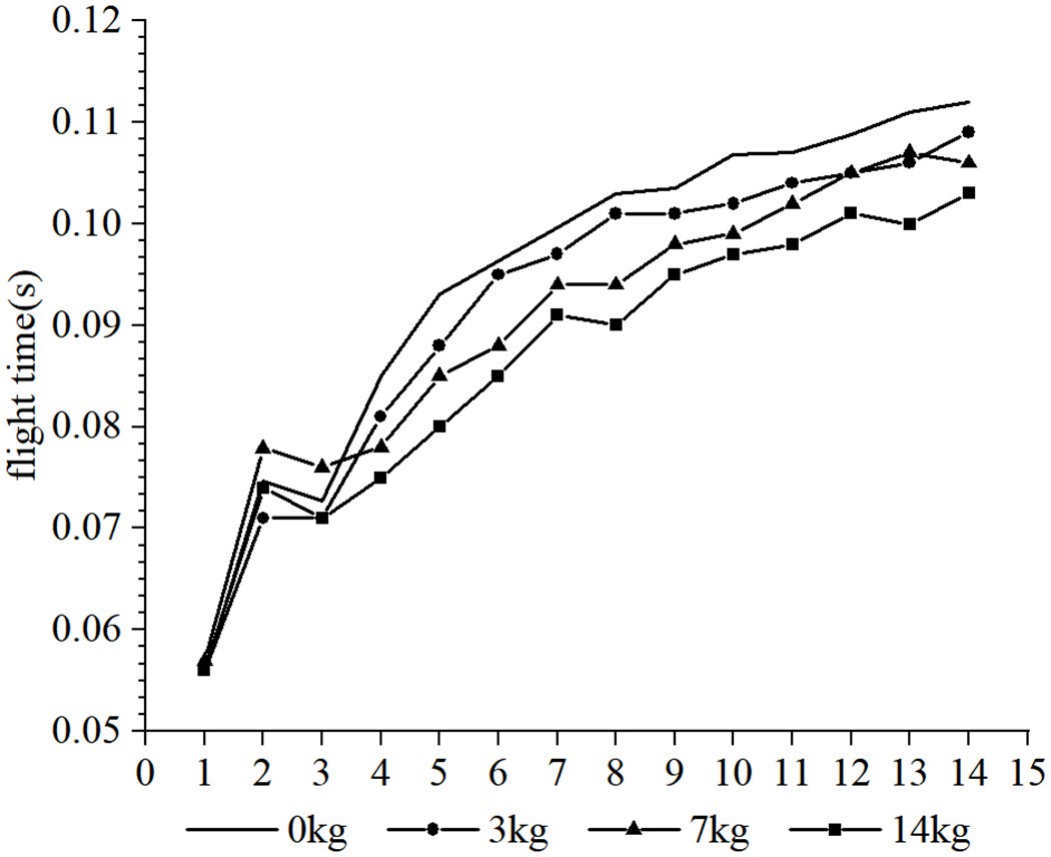
The effect of different resistance loads on flight time.

**Fig 9:**
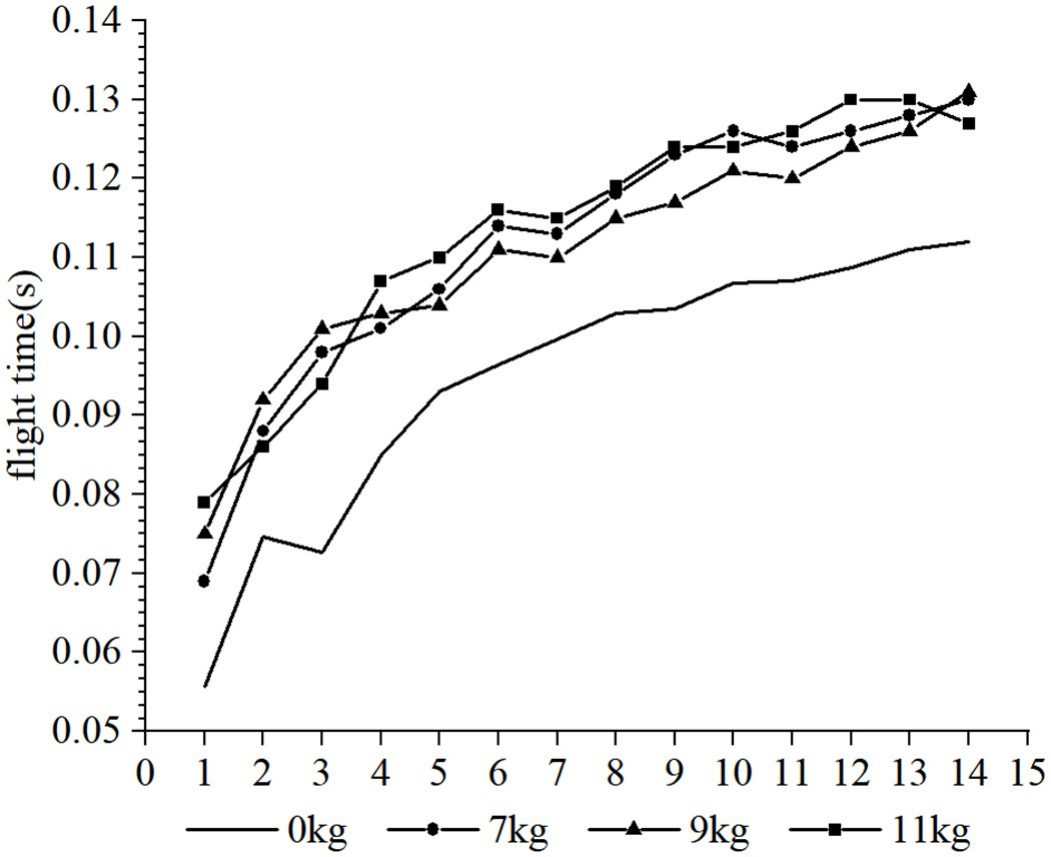
The effect of different assistance loads on flight time.

Furthermore, it was observed that the subjects took more steps to complete the 30-m sprint under the resistance conditions compared to the no-load condition, whereas fewer steps were taken under the assistance conditions. Due to the different loads and the differences in each participant’s physical qualities, such as strength, sprint technique and step length, different step sizes were produced over the 30-m distance. In order to conduct a cross-sectional analysis of the effect of different loads on the participants’ step-by-step sprint kinematics, data from the common first 14 steps were selected. Each variable examined was influenced to varying degrees by the different loading conditions. The step length tended to increase with the number of steps for each load condition (as shown in Figs 2 and 3, respectively), step time showed a tendency to decrease and stabilize (as shown in Figs 4 and 5, respectively), contact time tended to decrease (as shown in Figs 6 and 7, respectively), and flight time tended to increase (as shown in Figs 8 and 9, respectively). During the acceleration phase, the subject’s speed gradually increased, leading to a decrease in the horizontal force exerted and an increase in the vertical force exerted. Consequently, the contact time gradually decreased, and the flight time gradually increased. As the flight time and speed increased, the horizontal displacement of the subject during the flight also increased, resulting in a gradual increase in step length. As illustrated in Figs 2 and 3, under the resistance conditions, the step length for each step after the first step was shorter compared to the no-load condition. Conversely, under the assistance conditions, the step length for each step after the first step was longer compared to the no-load condition. The step length for each load condition differed significantly from the no-load condition (p<0.001). The effect sizes of the resistance conditions of 3 kg, 7 kg, and 14 kg on step length were small, moderate, and large (Cohen’s d = 0.19, 0.59, and 1.19), respectively. Similarly, the effect sizes of the 7 kg, 9 kg, and 11 kg assistance conditions on step length were moderate, large, and large (Cohen’s d = 0.68, 0.80, 0.96), respectively. As the resistance load increased, the subject’s displacement velocity during the flight phase decreased, resulting in a reduction in step length.

As shown in Figs 4 and 5, there is a tendency for the step time to decrease and then stabilize during the acceleration phase of the start, accompanied by a decrease and stabilization in step frequency. Both the resistance and assistance conditions result in an increase in step time of the subjects, and except for the 14 kg resistance condition, there is no significant difference in step time between the resistance and assistance condition groups. The step times under each load condition are significantly different from the step times under no-load conditions (p <0.001). The effect of the 3 kg, 7 kg, and 14 kg resistance conditions on step time is moderate, moderate, and large, respectively (Cohen’s d = 0.65, 0.71, and 1.18). The assistance conditions of 7 kg, 9 kg, and 11 kg all have a large effect on step time (Cohen’s d = 1.06, 0.82, 1.11). The increase in step time under resistance conditions is mainly due to an increase in contact time, while the increase in step time under assistance conditions is mainly due to an increase in flight time.

As shown in Figs 6 and 7, under the resisted condition, the subjects have longer contact times at each step than under the unloaded condition. Starting from step 4, the higher the load, the longer the contact time will be. However, under the assistance condition, the subjects have shorter contact times at each step after step 2 than under the unloaded condition. The effects of the resistance conditions of 3 kg, 7 kg, and 14 kg on contact time is small, small, and moderate (Cohen’s d = 0.24, 0.39, and 0.66), respectively. The effects of the assistance conditions of 7 kg, 9 kg and 11 kg on contact time are all small (Cohen’s d = 0.43, 0.48, 0.44). Under the resistance condition, the subjects needed to generate greater horizontal force and longer contact time to perform a sprint run against the load [10]. While under the assistance condition, the assistance force gives subjects the conditions to overspeed and complete the sprint acceleration within a shorter contact time.

As shown in Figs 8 and 9, under the resistance condition, there is a minimal difference in flight time for each step compared to the unloaded condition. In contrast, under the assistance condition, the flight time for each step is greater than the flight time under the unloaded condition. The flight times of the 3 kg and 7 kg resistance conditions do not show significant differences compared to the unloaded condition (p = 0.873, 0.141), while the flight time of the 14 kg resistance condition shows a significant difference but with a small effect (p = 0.006, Cohen’s d = 0.25). The flight times of the 7 kg, 9 kg, and 11 kg assistance conditions all show significant differences compared to the unloaded condition and have a large effect (p<0.001, Cohen’s d = 1.12, 1.16, 1.32). Both the resistance condition and the assistance condition with a given load have a small impact on the subjects’ technical movements in sprint. Under the resistance condition, the subjects generate greater horizontal force and have longer ground contact time to complete the acceleration, stimulating the muscles to generate sufficient force to sustain the flight phase. Under the assistance condition, due to an increased step length, the subjects need to adjust to a longer flight time to maintain sprint technique (contact position below the body’s center of gravity).

Different resistance and assistance loads have different degrees of influence on sprinting kinematics (Table 2).

**Table 2:** Effects of different load conditions on sprint kinematics.

	Resistance Load: 3kg	Resistance Load: 7kg	Resistance Load: 14kg	Assistance Load: 7kg	Assistance Load: 9kg	Assistance Load: 11kg
*Steplength*	p<0.001,Cohen’s d = 0.19	p<0.001,Cohen’s d = 0.59	p<0.001,Cohen’s d = 1.19	p<0.001,Cohen’s d = 0.68	p<0.001,Cohen’s d = 0.80	p<0.001,Cohen’s d = 0.96
*Steptime*	p <0.001,Cohen’s d = 0.65	p <0.001,Cohen’s d = 0.71	p <0.001,Cohen’s d = 1.18	p <0.001,Cohen’s d = 1.06	p <0.001,Cohen’s d = 0.82	p <0.001,Cohen’s d = 1.11
*Contacttime*	p<0.001,Cohen’s d = 0.24	p<0.001,Cohen’s d = 0.39	p<0.001,Cohen’s d = 0.66	p<0.001,Cohen’s d = 0.43	p<0.001,Cohen’s d = 0.48	p<0.001,Cohen’s d = 0.44
*Flighttime*	p = 0.873	p = 0.141	p = 0.006,Cohen’s d = 0.25	p<0.001,Cohen’s d = 1.12	p<0.001,Cohen’s d = 1.16	p<0.001,Cohen’s d = 1.32

## Discussion

Resistance running and assistance running are the two main types of training methods used in sprint. Kinematics is a visual representation of an athlete’s performance during running, therefore, it is crucial to study the effects of these training methods on sprinters’ kinematic data, taking into account not only time variations, but also focusing on changes in gait.

The results of the study demonstrate that most kinematics are influenced by resistance and assistance training, with the exception of the 3 kg and 7 kg resistance conditions, where no significant differences were observed compared to the unloaded condition. Notably, flight time showed a significant but small difference in the 14 kg resistance sprint condition compared to the unloaded condition. Analysis of the subjects’ kinematics under the resistance condition reveals that different load conditions have similar effects on step length, step time, and contact time. The magnitude of change in these gait data increases with the increasing load. However, the subjects’ flight times were not significantly affected by the resistance condition.

The findings suggest that under resistance loading, the lower limbs generate greater counter ground forces and longer contact times to maintain similar technical movements and flight times as in normal sprint conditions. Prior research suggests that when selecting resistance loads, the goal should be to reduce the athlete’s maximal velocity while minimizing the impact on sprint technique [31]. It is therefore necessary to test athletes’ maximal velocities under different resistance conditions to determine appropriate training loads [10, 26, 32, 33]. This approach ensures that the training load is optimized for effective results.

The analysis of the subjects’ gait parameters under the assistance conditions revealed that different loading conditions have similar effects on all kinematic parameters. However, the differences between the groups were not significant. This lack of significance may be attributed to the subjects’ active adjustments during the assistance-overdrive sprint to maintain body coordination, which limits their ability to achieve longer step lengths, shorter contact times, and longer flight times. Long-term systematic assistance training may enable athletes to learn necessary adjustments during the assisted sprint, allowing them to adapt to the overspeed condition. This adaptation can lead to an increase in step length while maintaining step frequency and an increase in flight time while reducing contact time [4]. Consequently, athletes can adjust their limbs during flight phase to minimize braking during the contact phase.

The analysis revealed that both the resistance and assistance conditions led to an increase in the subjects’ step time. Furthermore, the analysis of contact time and flight time indicated that the increase in step time under the resistance condition was primarily attributed to a significant increase in contact time, while the increase in step time under the assistance condition was primarily due to a significant increase in flight time. Consequently, the observed increase in 30-meter sprint time under the resistance condition can be attributed to a reduction in step length and an increase in step time. Conversely, the decrease in 30-meter sprint time under the assistance condition is a result of a significant increase in step length.

In summary, different loads result in variations in athletes’ kinematic parameters during sprint. The choice of load can significantly impact parameters such as step length, step time, contact time, and flight time. The magnitude of these changes may increase with heavier loads. Heavier resistance loads can temporarily reduce an athlete’s running speed, step length, and step frequency during training sessions. Over time, as athletes adapt to the loads, their performance is expected to improve. In contrast, lighter loads may not significantly affect an athlete’s strength and technique, but may help to translate the training effects of heavier loads into the ability to sprint unloaded. In assisted sprint, the use of different loads can affect athletes’ ability to maintain coordination and body posture. Assistance loads may require active adjustments from athletes to maintain technical movements. It is expected that long-term systematic assistance training enables adaptation to overspeed conditions, influencing step length, contact time, and flight time.

There are several limitations to this study. Firstly, the sample consisted only of males among college sprinters, which may limit the generalizability of sprint kinematics to different populations. Future research may benefit from a broader study involving participants of different genders and different sports. Additionally, in order to gain a more comprehensive understanding of athletes’ sprint ability, longitudinal studies are recommended to explore the combined effects of long-term systematic resistance and assistance training on sprint performance.

## Conclusion

The results of the study showed that compared to unloaded running, male collegiate sprinters increased their total steps count, decreased their step length, increased their step time, increased their contact time, and had little change in flight time when performing a 30-meter resisted sprint. Conversely, they decreased their total steps count, increased their step length, increased their step time, decreased their contact time, and increased their flight time when performing a 30-meter assisted sprint. The increase in 30-meter sprint time under resistance conditions was mainly due to a decrease in step length and an increase in step time, whereas the decrease in 30-meter sprint time under assistance conditions was due to a significant increase in step length. In addition, sprint kinematics were found to be more affected by the resisted sprint, with greater changes observed with increasing load. Under assistance conditions, there was no significant difference in the change of gait. Resistance sprint training can be used to increase the strength and explosive power of athletes during the initial phase, thereby improving their acceleration ability. However, it is important to note that long-term and high-frequency resisted sprint training may lead to a reduction in step length and step frequency in athletes. On the other hand, assisted sprint training can help reduce athletes’ contact time, improve their step length and flight capacity, and enable them to exceed their speed limits, thereby increasing their maximal speed capability.

## Supporting information

S1 Data (PDF)

S2 Data (PDF)

## References

[1] Beato M, Drust B, and Iacono AD. Implementing High-speed Running and Sprinting Training in Professional Soccer. Int J Sports Med. 2021; 42: 295–299. doi: 10.1055/a-1302-7968 33291180

[2] Spencer M, Bishop D, Dawson B, and Goodman C. Physiological and metabolic responses of repeated-sprint activities: specific to field-based team sports. Sports Med. 2005; 35: 1025–1044, 2005. doi: 10.2165/00007256-200535120-00003 16336007

[3] Clark KP, Stearne DJ, Walts CT, and Miller AD. The longitudinal effects of resisted sprint training using weighted sleds vs. weighted vests. J Strength Cond Res. 2010; 24: 3287–3295. doi: 10.1097/01.JSC.0000367084.42886.88 19996786

[4] Kristensen GO, van den Tillaar R, and Ettema GJ. Velocity specificity in early-phase sprint training. J Strength Cond Res. 2006; 20: 833–837. doi: 10.1519/00124278-200611000-00018 17194234

[5] Van den Tillaar R. Effect of different training programs on the velocity of overarm throwing: a brief review. J Strength Cond Res. 2004; 18: 388–396. doi: 10.1519/R-12792.1 15142008

[6] Mero A, Komi PV. Force-, EMG-, and elasticity-velocity relationships at submaximal, maximal and supramaximal running speeds in sprinters. Eur J Appl Physiol Occup Physiol. 1986; 55(5):553–561. doi: 10.1007/BF00421652 3769912

[7] Murphy AJ, Lockie RG, Coutts AJ. Kinematic determinants of early acceleration in field sport athletes. J Sports Sci Med. 2003; 2(4):144–50. 24688275 PMC3963247

[8] Cecilia-Gallego P, Odriozola A, Beltran-Garrido JV, and ´Alvarez-Herms J. Acute effects of overspeed stimuli with towing system on athletic sprint performance: A systematic review with meta-analysis. Journal of sports sciences. 2022; 40: 704–716. doi: 10.1080/02640414.2021.2015165 34991419

[9] Cronin J, Hansen K, Kawamori N, and McNair P. Effects of weighted vests and sled towing on sprint kinematics. Sports Biomech. 2008; 7: 160–172. doi: 10.1080/14763140701841381 18610770

[10] Alcaraz PE, Palao JM, Elvira JL, and Linthorne NP. Effects of three types of resisted sprint training devices on the kinematics of sprinting at maximum velocity. J Strength Cond Res. 2008; 22: 890–897. doi: 10.1519/JSC.0b013e31816611ea 18438225

[11] Paradisis GP and Cooke CB. Kinematic and postural characteristics of sprint running on sloping surfaces. J Sports Sci. 2001; 19: 149–159. doi: 10.1080/026404101300036370 11217013

[12] Hunter JP, Marshall RN, McNair PJ. Interaction of step length and step rate during sprint running. Med Sci Sports Exerc. 2004; 36(2): 261–271. doi: 10.1249/01.MSS.0000113664.15777.53 14767249

[13] Kawamori N, Newton RU, Hori N, and Nosaka K. Effects of weighted sled towing with heavy versus light load on sprint acceleration ability. J Strength Cond Res. 2014; 28: 2738–2745. doi: 10.1519/JSC.0b013e3182915ed4 23539079

[14] Kawamori N, Newton R, and Nosaka K. Effects of weighted sled towing on ground reaction force during the acceleration phase of sprint running. J Sports Sci. 2014; 32: 1139–1145. doi: 10.1080/02640414.2014.886129 24576071

[15] Matusi´nski A, Pietraszewski P, Krzysztofik M, and Go la´s A. The Effects of Resisted Post-Activation Sprint Performance Enhancement in Elite Female Sprinters. Frontiers in physiology. 2021; 12: 651659. doi: 10.3389/fphys.2021.651659 33746784 PMC7973236

[16] Matusi´nski A, Go las A, Zajac A, and Maszczyk A. Acute effects of resisted and assisted locomotor activation on sprint performance. Biology of sport. 2022; 39: 1049–1054. doi: 10.5114/biolsport.2022.108706 36247959 PMC9536394

[17] Moya-Ramon M, Nakamura FY, Teixeira AS, Granacher U, Santos-Rosa FJ, Sanz-Rivas D, et al. Effects of Resisted Vs. Conventional Sprint Training on Physical Fitness in Young Elite Tennis Players. Journal of human kinetics. 2020; 73: 181–192. doi: 10.2478/hukin-2019-0142 32774549 PMC7386135

[18] Kawamori N, Newton R, and Nosaka K. Effects of weighted sled towing on ground reaction force during the acceleration phase of sprint running. Journal of human kinetics. 2020; 73: 181–192. 24576071 10.1080/02640414.2014.886129

[19] Clark DA, Sabick MB, Pfeiffer RP, Kuhlman SM, Knigge NA, and Shea KG. Influence of towing force magnitude on the kinematics of supramaximal sprinting. J Strength Cond Res. 2009; 23: 1162–1168. doi: 10.1519/JSC.0b013e318194df84 19528855

[20] Lahti J, Jim´enez-Reyes P, Cross MR, Samozino P, Chassaing P, Simond-Cote B, et al. Individual Sprint Force-Velocity Profile Adaptations to In-Season Assisted and Resisted Velocity-Based Training in Professional Rugby. Sports (Basel, Switzerland). 2020; 8. doi: 10.3390/sports8050074 32466235 PMC7281595

[21] van den Tillaar R and Gamble P. Comparison of step-by-step kinematics of resisted, assisted and unloaded 20-m sprint runs. Sports Biomech. 2019;18: 539–552. doi: 10.1080/14763141.2018.1442871 29578385

[22] van den Tillaar R. Comparison of development of step-kinematics of assisted 60m sprints with different pulling forces between experienced male and female sprinters. PloS one. 2021; 16: e0255302. doi: 10.1371/journal.pone.0255302 34314453 PMC8315524

[23] Mangine GT, Huet K, Williamson C, Bechke E, Serafini P, Bender D, et al. A Resisted Sprint Improves Rate of Force Development During a 20-m Sprint in Athletes. J Strength Cond Res. 2018; 32: 1531–1537. doi: 10.1519/JSC.0000000000002030 29786621

[24] Rakovic E, Paulsen G, Helland C, Eriksrud O, and Haugen T. The effect of individualised sprint training in elite female team sport athletes: A pilot study. J Sports Sci. 2018; 36: 2802–2808. doi: 10.1080/02640414.2018.1474536 29741443

[25] Cross MR, Lahti J, Brown SR, Chedati M, Jimenez-Reyes P, Samozino P, et al. Training at maximal power in resisted sprinting: Optimal load determination methodology and pilot results in team sport athletes. PloS one. 2018; 13: e0195477. doi: 10.1371/journal.pone.0195477 29641589 PMC5895020

[26] Alcaraz PE, Palao JM, and Elvira JL. Determining the optimal load for resisted sprint training with sled towing. J Strength Cond Res. 2009; 23: 480–485. doi: 10.1519/JSC.0b013e318198f92c 19197200

[27] Cottle CA, Carlson LA, and Lawrence MA. Effects of sled towing on sprint starts. J Strength Cond Res. 2014; 28: 1241–1245. doi: 10.1519/JSC.0000000000000396 24513621

[28] Grgic J, Lazinica B, Garofolini A, Schoenfeld BJ, Saner NJ, and Mikulic P. The effects of time of day-specific resistance training on adaptations in skeletal muscle hypertrophy and muscle strength: A systematic review and meta-analysis. Chronobiol Int. 2019; 36: 449–460. doi: 10.1080/07420528.2019.1567524 30704301

[29] Moir G, Button C, Glaister M, and Stone MH. Influence of familiarization on the reliability of vertical jump and acceleration sprinting performance in physically active men. J Strength Cond Res. 2004; 18: 276–280. doi: 10.1519/00124278-200405000-00013 15142028

[30] Morin JB, Slawinski J, Dorel S, de Villareal ES, Couturier A, Samozino P, et al. Acceleration capability in elite sprinters and ground impulse: Push more, brake less?. J Biomech. 2015; 48: 3149–3154. doi: 10.1016/j.jbiomech.2015.07.009 26209876

[31] Alcaraz PE, Carlos-Vivas J, Oponjuru BO, and Martinez-Rodriguez A. The effectiveness of resisted sled training (RST) for sprint performance: a systematic review and meta-analysis. Sports Medicine. 2018; 48: 2143–2165. doi: 10.1007/s40279-018-0957-6 29926369

[32] Lockie RG, Murphy AJ, and Spinks CD. Effects of resisted sled towing on sprint kinematics in field-sport athletes. J Strength Cond Res. 2003; 17: 760–767. doi: 10.1519/1533-4287(2003)017<0760:EORSTO>2.0.CO;2 14636109

[33] Maulder PS, Bradshaw EJ, and Keogh JW. Kinematic alterations due to different loading schemes in early acceleration sprint performance from starting blocks. J Strength Cond Res. 2008; 22: 1992–2002. doi: 10.1519/JSC.0b013e31818746fe 18978610

