# Supplementary material for: The effect of different resistance and assistance loads on 30-m sprint kinematics
Source: PLoS One. 2024 Mar 22;19(3):e0298517. doi: 10.1371/journal.pone.0298517 (PMC10959337; doi:10.1371/journal.pone.0298517)
Supplement: S1 Data — (PDF) [file pone.0298517.s001.pdf]

0 kg time

0-10m	10-20m	20-30m
1.66	1.27	1.21
1.68	1.31	1.21
1.62	1.24	1.2
1.65	1.28	1.22
1.65	1.26	1.17
1.65	1.32	1.22
1.65	1.25	1.19
1.66	1.31	1.22
1.64	1.29	1.2
1.64	1.25	1.16
1.6	1.17	1.1
1.68	1.31	1.21
1.65	1.3	1.23
1.66	1.29	1.23

3kg resistance time

0-10m	10-20m	20-30m
1.98	1.33	1.31
2	1.37	1.28
1.96	1.31	1.25
2.01	1.36	1.26
2	1.37	1.32
2	1.4	1.33
1.97	1.32	1.24
1.99	1.37	1.25
1.97	1.32	1.26
1.98	1.38	1.28
1.97	1.29	1.19
2.01	1.41	1.32
2	1.4	1.3
1.99	1.36	1.3

7kg resistance time

0-10m	10-20m	20-30m
2.19	1.41	1.34
2.22	1.42	1.35
2.19	1.4	1.36
2.3	1.52	1.4
2.25	1.47	1.39
2.24	1.45	1.39
2.25	1.47	1.41
2.24	1.45	1.39
2.26	1.55	1.52
2.2	1.41	1.32
2.2	1.37	1.29
2.24	1.44	1.37
2.24	1.47	1.39
2.23	1.45	1.35

14kg resistance time

0-10m	10-20m	20-30m
2.43	1.67	1.74
2.49	1.81	1.82
2.42	1.65	1.68
2.47	1.71	1.7
2.4	1.62	1.62
2.47	1.77	1.75
2.45	1.71	1.7
2.53	1.79	1.76
2.44	1.74	1.74
2.44	1.71	1.7
2.41	1.62	1.55
2.52	1.8	1.75
2.43	1.68	1.65
2.43	1.6	1.63

7kg assistance time

0-10m	10-20m	20-30m
1.51	1.18	1.09
1.5	1.18	1.05
1.52	1.2	1.11
1.52	1.22	1.09
1.53	1.22	1.16
1.54	1.25	1.17
1.49	1.15	1.03
1.5	1.21	1.12
1.49	1.2	1.11
1.5	1.19	1.06
1.46	1.09	0.98
1.53	1.2	1.08
1.5	1.19	1.08
1.52	1.22	1.12

9kg assistance time

0-10m	10-20m	20-30m
1.48	1.12	1.02
1.49	1.13	1.03
1.5	1.16	1.06
1.53	1.22	1.1
1.52	1.18	1.1
1.55	1.24	1.14
1.52	1.13	1.059
1.48	1.16	1.03
1.49	1.16	1.05
1.48	1.13	1.01
1.46	1.11	0.98
1.52	1.18	1.06
1.51	1.18	1.09
1.48	1.17	1.08

11kg assistance time

0-10m	10-20m	20-30m
1.47	1.14	1.01
1.49	1.17	1.05
1.5	1.17	1.04
1.52	1.19	1.08
1.53	1.22	1.12
1.48	1.16	1.03
1.47	1.13	1.03
1.49	1.15	1.04
1.5	1.15	1.04
1.46	1.08	0.98
1.47	1.12	1
1.48	1.13	1.04
1.48	1.14	1.02
1.49	1.16	1.04
