# Supplementary material for: The effect of different resistance and assistance loads on 30-m sprint kinematics
Source: PLoS One. 2024 Mar 22;19(3):e0298517. doi: 10.1371/journal.pone.0298517 (PMC10959337; doi:10.1371/journal.pone.0298517)
Supplement: S2 Data — (PDF) [file pone.0298517.s002.pdf]

| | 0kg | | | 3kg resistance | | |
|---|---|---|---|---|---|---|
| step | step length | flight time | contact time | step length | flight time | contact time |
| 1 | 1.37 | 0.070 | 0.158 | 1.34 | 0.070 | 0.170 |
| 2 | 1.06 | 0.075 | 0.170 | 0.93 | 0.041 | 0.179 |
| 3 | 1.54 | 0.083 | 0.129 | 1.36 | 0.079 | 0.145 |
| 4 | 1.39 | 0.091 | 0.137 | 1.24 | 0.091 | 0.137 |
| 5 | 1.63 | 0.087 | 0.125 | 1.54 | 0.087 | 0.133 |
| 6 | 1.59 | 0.100 | 0.125 | 1.45 | 0.104 | 0.129 |
| 7 | 1.81 | 0.108 | 0.116 | 1.61 | 0.095 | 0.120 |
| 8 | 1.77 | 0.100 | 0.116 | 1.55 | 0.104 | 0.125 |
| 9 | 1.86 | 0.112 | 0.116 | 1.65 | 0.095 | 0.120 |
| 10 | 1.91 | 0.120 | 0.112 | 1.65 | 0.104 | 0.125 |
| 11 | 1.93 | 0.112 | 0.112 | 1.68 | 0.104 | 0.112 |
| 12 | 1.99 | 0.112 | 0.104 | 1.73 | 0.104 | 0.116 |
| 13 | 2.03 | 0.125 | 0.112 | 1.69 | 0.091 | 0.116 |
| 14 | 2.12 | 0.125 | 0.100 | 1.84 | 0.112 | 0.120 |

| | 0kg | | | 3kg resistance | | |
|---|---|---|---|---|---|---|
| step | step length | flight time | contact time | step length | flight time | contact time |
| 1 | 1.21 | 0.045 | 0.158 | 0.80 | 0.054 | 0.220 |
| 2 | 0.92 | 0.091 | 0.154 | 1.22 | 0.075 | 0.158 |
| 3 | 1.42 | 0.075 | 0.129 | 1.06 | 0.062 | 0.158 |
| 4 | 1.23 | 0.091 | 0.137 | 1.42 | 0.079 | 0.145 |
| 5 | 1.50 | 0.079 | 0.129 | 1.39 | 0.095 | 0.141 |
| 6 | 1.50 | 0.091 | 0.129 | 1.54 | 0.100 | 0.137 |
| 7 | 1.64 | 0.087 | 0.133 | 1.60 | 0.087 | 0.129 |
| 8 | 1.63 | 0.100 | 0.129 | 1.68 | 0.095 | 0.129 |
| 9 | 1.71 | 0.104 | 0.129 | 1.65 | 0.100 | 0.133 |
| 10 | 1.73 | 0.100 | 0.116 | 1.72 | 0.095 | 0.129 |
| 11 | 1.77 | 0.116 | 0.120 | 1.78 | 0.095 | 0.129 |
| 12 | 1.79 | 0.108 | 0.116 | 1.87 | 0.104 | 0.125 |
| 13 | 1.77 | 0.112 | 0.166 | 1.79 | 0.116 | 0.125 |
| 14 | 1.91 | 0.125 | 0.112 | 1.90 | 0.108 | 0.114 |

| | 0kg | | | 3kg resistance | | |
|---|---|---|---|---|---|---|
| step | step length | flight time | contact time | step length | flight time | contact time |
| 1 | 1.13 | 0.037 | 0.216 | 0.78 | 0.050 | 0.212 |
| 2 | 1.55 | 0.075 | 0.175 | 1.20 | 0.070 | 0.154 |
| 3 | 1.52 | 0.062 | 0.150 | 1.14 | 0.054 | 0.154 |
| 4 | 1.77 | 0.062 | 0.158 | 1.40 | 0.070 | 0.162 |
| 5 | 1.71 | 0.095 | 0.150 | 1.34 | 0.095 | 0.137 |
| 6 | 1.92 | 0.083 | 0.125 | 1.54 | 0.087 | 0.129 |
| 7 | 1.86 | 0.100 | 0.129 | 1.47 | 0.095 | 0.133 |
| 8 | 1.95 | 0.091 | 0.125 | 1.57 | 0.100 | 0.120 |
| 9 | 2.06 | 0.100 | 0.120 | 1.58 | 0.095 | 0.116 |

| | | | | | | |
|---|---|---|---|---|---|---|
| 10 | 2.08 | 0.116 | 0.116 | 1.66 | 0.087 | 0.129 |
| 11 | 2.07 | 0.100 | 0.108 | 1.73 | 0.112 | 0.125 |
| 12 | 2.13 | 0.104 | 0.116 | 1.64 | 0.104 | 0.112 |
| 13 | 2.40 | 0.120 | 0.112 | 1.74 | 0.108 | 0.116 |
| 14 | | | | | | |

| | 0kg | | | 3kg resistance | | |
|---|---|---|---|---|---|---|
| step | step length | flight time | contact time | step length | flight time | contact time |
| 1 | 1.17 | 0.045 | 0.175 | 1.24 | 0.066 | 0.195 |
| 2 | 1.01 | 0.058 | 0.162 | 0.95 | 0.087 | 0.162 |
| 3 | 1.43 | 0.083 | 0.170 | 1.33 | 0.083 | 0.133 |
| 4 | 1.26 | 0.091 | 0.133 | 1.19 | 0.083 | 0.141 |
| 5 | 1.58 | 0.083 | 0.129 | 1.51 | 0.091 | 0.137 |
| 6 | 1.50 | 0.100 | 0.137 | 1.45 | 0.104 | 0.129 |
| 7 | 1.69 | 0.104 | 0.108 | 1.75 | 0.125 | 0.120 |
| 8 | 1.66 | 0.095 | 0.129 | 1.60 | 0.116 | 0.116 |
| 9 | 1.80 | 0.104 | 0.112 | 1.70 | 0.108 | 0.120 |
| 10 | 1.78 | 0.116 | 0.120 | 1.66 | 0.116 | 0.108 |
| 11 | 1.83 | 0.108 | 0.104 | 1.78 | 0.133 | 0.108 |
| 12 | 1.94 | 0.125 | 0.112 | 1.77 | 0.125 | 0.100 |
| 13 | 1.89 | 0.116 | 0.108 | 1.81 | 0.120 | 0.104 |
| 14 | 2.03 | 0.125 | 0.104 | 1.88 | 0.133 | 0.112 |

| | 0kg | | | 3kg resistance | | |
|---|---|---|---|---|---|---|
| step | step length | flight time | contact time | step length | flight time | contact time |
| 1 | 1.03 | 0.054 | 0.212 | 0.96 | 0.058 | 0.225 |
| 2 | 1.38 | 0.070 | 0.179 | 1.40 | 0.079 | 0.170 |
| 3 | 1.19 | 0.058 | 0.158 | 1.14 | 0.070 | 0.158 |
| 4 | 1.53 | 0.070 | 0.162 | 1.47 | 0.070 | 0.158 |
| 5 | 1.41 | 0.070 | 0.150 | 1.38 | 0.091 | 0.145 |
| 6 | 1.62 | 0.087 | 0.150 | 1.58 | 0.095 | 0.133 |
| 7 | 1.47 | 0.087 | 0.150 | 1.55 | 0.095 | 0.133 |
| 8 | 1.64 | 0.087 | 0.129 | 1.65 | 0.112 | 0.129 |
| 9 | 1.60 | 0.083 | 0.137 | 1.66 | 0.108 | 0.120 |
| 10 | 1.69 | 0.108 | 0.125 | 1.66 | 0.112 | 0.125 |
| 11 | 1.65 | 0.100 | 0.120 | 1.70 | 0.108 | 0.120 |
| 12 | 1.69 | 0.095 | 0.116 | 1.65 | 0.095 | 0.125 |
| 13 | 1.75 | 0.108 | 0.125 | 1.75 | 0.112 | 0.125 |
| 14 | 1.74 | 0.125 | 0.125 | 1.69 | 0.112 | 0.125 |

| | 0kg | | | 3kg resistance | | |
|---|---|---|---|---|---|---|
| step | step length | flight time | contact time | step length | flight time | contact time |
| 1 | 0.64 | 0.058 | 0.191 | 1.10 | 0.058 | 0.179 |
| 2 | 1.14 | 0.054 | 0.141 | 0.81 | 0.066 | 0.154 |
| 3 | 0.81 | 0.066 | 0.133 | 1.24 | 0.070 | 0.133 |
| 4 | 1.29 | 0.058 | 0.129 | 0.99 | 0.062 | 0.125 |

| | | | | | | |
|---|---|---|---|---|---|---|
| 5 | 1.15 | 0.066 | 0.137 | 1.45 | 0.079 | 0.137 |
| 6 | 1.54 | 0.079 | 0.129 | 1.26 | 0.087 | 0.120 |
| 7 | 1.42 | 0.087 | 0.120 | 1.52 | 0.095 | 0.112 |
| 8 | 1.66 | 0.091 | 0.116 | 1.44 | 0.108 | 0.100 |
| 9 | 1.45 | 0.079 | 0.112 | 1.59 | 0.095 | 0.116 |
| 10 | 1.73 | 0.087 | 0.112 | 1.57 | 0.095 | 0.116 |
| 11 | 1.81 | 0.083 | 0.108 | 1.56 | 0.091 | 0.104 |
| 12 | 1.96 | 0.091 | 0.125 | 1.71 | 0.095 | 0.120 |
| 13 | 2.14 | 0.095 | 0.112 | 1.66 | 0.100 | 0.116 |
| 14 | 2.12 | 0.104 | 0.116 | 1.84 | 0.112 | 0.116 |

| | 0kg | | | 3kg resistance | | |
|---|---|---|---|---|---|---|
| step | step length | flight time | contact time | step length | flight time | contact time |
| 1 | 0.76 | 0.054 | 0.175 | 1.29 | 0.058 | 0.179 |
| 2 | 1.32 | 0.062 | 0.150 | 1.02 | 0.066 | 0.179 |
| 3 | 1.14 | 0.062 | 0.154 | 1.36 | 0.087 | 0.145 |
| 4 | 1.47 | 0.066 | 0.141 | 1.31 | 0.087 | 0.133 |
| 5 | 1.37 | 0.079 | 0.133 | 1.51 | 0.087 | 0.133 |
| 6 | 1.52 | 0.083 | 0.133 | 1.46 | 0.108 | 0.120 |
| 7 | 1.47 | 0.083 | 0.116 | 1.62 | 0.104 | 0.112 |
| 8 | 1.53 | 0.083 | 0.125 | 1.57 | 0.100 | 0.120 |
| 9 | 1.55 | 0.087 | 0.116 | 1.66 | 0.104 | 0.112 |
| 10 | 1.55 | 0.091 | 0.120 | 1.68 | 0.108 | 0.112 |
| 11 | 1.61 | 0.095 | 0.108 | 1.66 | 0.104 | 0.104 |
| 12 | 1.56 | 0.095 | 0.116 | 1.74 | 0.108 | 0.108 |
| 13 | 1.71 | 0.095 | 0.112 | 1.69 | 0.108 | 0.104 |
| 14 | 1.57 | 0.104 | 0.112 | 1.85 | 0.100 | 0.108 |

| | 0kg | | | 3kg resistance | | |
|---|---|---|---|---|---|---|
| step | step length | flight time | contact time | step length | flight time | contact time |
| 1 | 0.72 | 0.058 | 0.229 | 1.32 | 0.070 | 0.216 |
| 2 | 1.45 | 0.066 | 0.166 | 1.07 | 0.075 | 0.175 |
| 3 | 1.12 | 0.095 | 0.166 | 1.40 | 0.075 | 0.166 |
| 4 | 1.57 | 0.066 | 0.133 | 1.31 | 0.079 | 0.150 |
| 5 | 1.38 | 0.079 | 0.154 | 1.54 | 0.087 | 0.154 |
| 6 | 1.75 | 0.066 | 0.145 | 1.54 | 0.075 | 0.141 |
| 7 | 1.68 | 0.087 | 0.150 | 1.70 | 0.112 | 0.145 |
| 8 | 1.86 | 0.070 | 0.129 | 1.66 | 0.095 | 0.129 |
| 9 | 2.02 | 0.095 | 0.141 | 1.74 | 0.108 | 0.133 |
| 10 | 2.19 | 0.087 | 0.125 | 1.71 | 0.087 | 0.125 |
| 11 | 2.42 | 0.112 | 0.133 | 1.81 | 0.116 | 0.133 |
| 12 | 2.37 | 0.095 | 0.125 | 1.88 | 0.087 | 0.129 |
| 13 | 2.65 | 0.112 | 0.120 | 1.81 | 0.116 | 0.133 |
| 14 | 2.52 | 0.091 | 0.120 | 1.98 | 0.112 | 0.120 |

| | 0kg | | | 3kg resistance | | |
|---|---|---|---|---|---|---|
| step | step length | flight time | contact time | step length | flight time | contact time |
| 1 | 1.36 | 0.050 | 0.216 | 1.28 | 0.058 | 0.204 |
| 2 | 1.04 | 0.054 | 0.191 | 1.03 | 0.066 | 0.179 |
| 3 | 1.52 | 0.066 | 0.166 | 1.43 | 0.079 | 0.170 |
| 4 | 1.29 | 0.075 | 0.162 | 1.28 | 0.087 | 0.158 |
| 5 | 1.67 | 0.091 | 0.137 | 1.52 | 0.100 | 0.145 |
| 6 | 1.57 | 0.095 | 0.141 | 1.49 | 0.091 | 0.137 |
| 7 | 1.71 | 0.091 | 0.133 | 1.61 | 0.087 | 0.137 |
| 8 | 1.69 | 0.087 | 0.137 | 1.64 | 0.091 | 0.141 |
| 9 | 1.82 | 0.100 | 0.133 | 1.70 | 0.100 | 0.133 |
| 10 | 1.84 | 0.104 | 0.129 | 1.75 | 0.100 | 0.129 |
| 11 | 1.85 | 0.104 | 0.120 | 1.75 | 0.091 | 0.141 |
| 12 | 1.93 | 0.104 | 0.125 | 1.85 | 0.095 | 0.137 |
| 13 | 1.85 | 0.100 | 0.120 | 1.79 | 0.100 | 0.125 |
| 14 | 2.04 | 0.104 | 0.125 | 2.00 | 0.104 | 0.137 |

| | 0kg | | | 3kg resistance | | |
|---|---|---|---|---|---|---|
| step | step length | flight time | contact time | step length | flight time | contact time |
| 1 | 0.89 | 0.033 | 0.208 | 1.15 | 0.058 | 0.154 |
| 2 | 1.36 | 0.062 | 0.191 | 0.81 | 0.079 | 0.162 |
| 3 | 1.00 | 0.045 | 0.158 | 1.26 | 0.058 | 0.137 |
| 4 | 1.42 | 0.062 | 0.162 | 1.09 | 0.066 | 0.158 |
| 5 | 1.24 | 0.083 | 0.141 | 1.44 | 0.075 | 0.145 |
| 6 | 1.49 | 0.100 | 0.129 | 1.22 | 0.083 | 0.133 |
| 7 | 1.45 | 0.087 | 0.116 | 1.52 | 0.091 | 0.125 |
| 8 | 1.52 | 0.095 | 0.125 | 1.47 | 0.095 | 0.125 |
| 9 | 1.53 | 0.100 | 0.120 | 1.58 | 0.087 | 0.125 |
| 10 | 1.55 | 0.100 | 0.108 | 1.59 | 0.116 | 0.125 |
| 11 | 1.63 | 0.100 | 0.112 | 1.64 | 0.091 | 0.108 |
| 12 | 1.57 | 0.112 | 0.108 | 1.73 | 0.112 | 0.125 |
| 13 | 1.74 | 0.125 | 0.095 | 1.69 | 0.108 | 0.120 |
| 14 | 1.60 | 0.112 | 0.104 | 1.71 | 0.100 | 0.116 |

| | 0kg | | | 3kg resistance | | |
|---|---|---|---|---|---|---|
| step | step length | flight time | contact time | step length | flight time | contact time |
| 1 | 1.42 | 0.100 | 0.170 | 0.87 | 0.037 | 0.170 |
| 2 | 1.24 | 0.100 | 0.141 | 1.62 | 0.100 | 0.170 |
| 3 | 1.60 | 0.087 | 0.137 | 1.36 | 0.087 | 0.137 |
| 4 | 1.44 | 0.116 | 0.125 | 1.79 | 0.112 | 0.133 |
| 5 | 1.69 | 0.095 | 0.120 | 1.67 | 0.104 | 0.112 |
| 6 | 1.67 | 0.108 | 0.120 | 1.91 | 0.120 | 0.116 |
| 7 | 1.82 | 0.104 | 0.120 | 1.86 | 0.108 | 0.112 |
| 8 | 1.75 | 0.116 | 0.116 | 1.98 | 0.104 | 0.116 |
| 9 | 1.75 | 0.108 | 0.108 | 2.05 | 0.129 | 0.112 |

| | | | | | | |
|---|---|---|---|---|---|---|
| 10 | 1.82 | 0.129 | 0.095 | 1.94 | 0.116 | 0.108 |
| 11 | 1.68 | 0.095 | 0.108 | 2.12 | 0.112 | 0.100 |
| 12 | 1.82 | 0.120 | 0.100 | 2.13 | 0.133 | 0.108 |
| 13 | 1.70 | 0.104 | 0.100 | 2.19 | 0.108 | 0.100 |
| 14 | 1.89 | 0.125 | 0.104 | 2.01 | 0.116 | 0.104 |

| | 0kg | | | 3kg resistance | | |
|---|---|---|---|---|---|---|
| step | step length | flight time | contact time | step length | flight time | contact time |
| 1 | 0.57 | 0.029 | 0.229 | 1.13 | 0.037 | 0.195 |
| 2 | 1.33 | 0.070 | 0.162 | 1.41 | 0.058 | 0.237 |
| 3 | 0.94 | 0.057 | 0.170 | 1.15 | 0.050 | 0.170 |
| 4 | 1.39 | 0.075 | 0.150 | 1.56 | 0.083 | 0.187 |
| 5 | 1.28 | 0.083 | 0.141 | 1.33 | 0.070 | 0.150 |
| 6 | 1.50 | 0.091 | 0.137 | 1.71 | 0.100 | 0.162 |
| 7 | 1.43 | 0.079 | 0.129 | 1.59 | 0.087 | 0.141 |
| 8 | 1.59 | 0.091 | 0.129 | 1.67 | 0.108 | 0.158 |
| 9 | 1.54 | 0.091 | 0.129 | 1.73 | 0.095 | 0.129 |
| 10 | 1.67 | 0.104 | 0.129 | 1.67 | 0.108 | 0.150 |
| 11 | 1.68 | 0.095 | 0.129 | 1.73 | 0.100 | 0.129 |
| 12 | 1.69 | 0.104 | 0.116 | 1.67 | 0.120 | 0.137 |
| 13 | 1.74 | 0.100 | 0.125 | 1.81 | 0.091 | 0.125 |
| 14 | 1.75 | 0.108 | 0.120 | 1.67 | 0.108 | 0.137 |

| | 0kg | | | 3kg resistance | | |
|---|---|---|---|---|---|---|
| step | step length | flight time | contact time | step length | flight time | contact time |
| 1 | 0.87 | 0.050 | 0.170 | 0.87 | 0.0.75 | 0.229 |
| 2 | 1.20 | 0.083 | 0.145 | 1.34 | 0.066 | 0.204 |
| 3 | 1.13 | 0.070 | 0.158 | 1.11 | 0.070 | 0.183 |
| 4 | 1.45 | 0.108 | 0.125 | 1.48 | 0.091 | 0.179 |
| 5 | 1.41 | 0.083 | 0.137 | 1.21 | 0.095 | 0.150 |
| 6 | 1.69 | 0.100 | 0.145 | 1.57 | 0.083 | 0.162 |
| 7 | 1.78 | 0.100 | 0.141 | 1.60 | 0.091 | 0.158 |
| 8 | 1.69 | 0.116 | 0.125 | 1.71 | 0.091 | 0.162 |
| 9 | 1.80 | 0.112 | 0.120 | 1.70 | 0.100 | 0.145 |
| 10 | 1.94 | 0.137 | 0.116 | 1.86 | 0.087 | 0.154 |
| 11 | 1.92 | 0.112 | 0.133 | 2.01 | 0.100 | 0.158 |
| 12 | 1.93 | 0.120 | 0.120 | 1.94 | 0.091 | 0.158 |
| 13 | 1.99 | 0.120 | 0.125 | 2.11 | 0.100 | 0.158 |
| 14 | 2.01 | 0.141 | 0.116 | 2.06 | 0.100 | 0.162 |

| | 0kg | | | 3kg resistance | | |
|---|---|---|---|---|---|---|
| step | step length | flight time | contact time | step length | flight time | contact time |
| 1 | 1.30 | 0.083 | 0.166 | 1.08 | 0.056 | 0.191 |
| 2 | 1.16 | 0.112 | 0.154 | 1.22 | 0.075 | 0.163 |
| 3 | 1.44 | 0.095 | 0.129 | 1.30 | 0.073 | 0.150 |

| | | | | | | |
|---|---|---|---|---|---|---|
| 4 | 1.50 | 0.120 | 0.125 | 1.43 | 0.085 | 0.141 |
| 5 | 1.65 | 0.100 | 0.120 | 1.49 | 0.095 | 0.136 |
| 6 | 1.71 | 0.125 | 0.129 | 1.60 | 0.096 | 0.133 |
| 7 | 1.70 | 0.108 | 0.116 | 1.63 | 0.100 | 0.127 |
| 8 | 1.84 | 0.120 | 0.116 | 1.69 | 0.103 | 0.124 |
| 9 | 1.84 | 0.108 | 0.116 | 1.72 | 0.103 | 0.122 |
| 10 | 1.98 | 0.125 | 0.108 | 1.79 | 0.107 | 0.116 |
| 11 | 1.94 | 0.120 | 0.116 | 1.80 | 0.106 | 0.116 |
| 12 | 2.07 | 0.125 | 0.108 | 1.85 | 0.109 | 0.114 |
| 13 | 1.96 | 0.108 | 0.116 | 1.89 | 0.110 | 0.117 |
| 14 | 2.16 | 0.125 | 0.112 | 1.92 | 0.116 | 0.113 |

7kg resistance

| step length | flight time | contact time |
|---|---|---|
| 1.26 | 0.062 | 0.187 |
| 1.03 | 0.104 | 0.166 |
| 1.35 | 0.083 | 0.137 |
| 1.15 | 0.095 | 0.145 |
| 1.53 | 0.083 | 0.120 |
| 1.42 | 0.104 | 0.137 |
| 1.51 | 0.108 | 0.112 |
| 1.48 | 0.108 | 0.116 |
| 1.64 | 0.100 | 0.116 |
| 1.59 | 0.095 | 0.120 |
| 1.64 | 0.095 | 0.120 |
| 1.75 | 0.108 | 0.120 |
| 1.73 | 0.108 | 0.116 |
| 1.76 | 0.104 | 0.112 |

14kg resistance

| step length | flight time | contact time |
|---|---|---|
| 1.21 | 0.087 | 0.133 |
| 0.80 | 0.062 | 0.179 |
| 1.32 | 0.075 | 0.145 |
| 1.10 | 0.079 | 0.179 |
| 1.40 | 0.091 | 0.133 |
| 1.26 | 0.100 | 0.150 |
| 1.45 | 0.095 | 0.125 |
| 1.36 | 0.104 | 0.141 |
| 1.46 | 0.108 | 0.133 |
| 1.41 | 0.100 | 0.141 |
| 1.46 | 0.091 | 0.120 |
| 1.49 | 0.112 | 0.154 |
| 1.53 | 0.104 | 0.120 |
| 1.59 | 0.108 | 0.145 |

7kg resistance

| step length | flight time | contact time |
|---|---|---|
| 0.87 | 0.057 | 0.216 |
| 1.17 | 0.062 | 0.179 |
| 0.97 | 0.083 | 0.162 |
| 1.28 | 0.087 | 0.125 |
| 1.19 | 0.112 | 0.141 |
| 1.40 | 0.091 | 0.125 |
| 1.28 | 0.104 | 0.133 |
| 1.43 | 0.108 | 0.129 |
| 1.42 | 0.125 | 0.116 |
| 1.43 | 0.100 | 0.125 |
| 1.48 | 0.112 | 0.120 |
| 1.47 | 0.100 | 0.125 |
| 1.47 | 0.108 | 0.129 |
| 1.56 | 0.112 | 0.125 |

14kg resistance

| step length | flight time | contact time |
|---|---|---|
| 0.84 | 0.062 | 0.220 |
| 1.00 | 0.054 | 0.195 |
| 0.99 | 0.066 | 0.175 |
| 1.25 | 0.062 | 0.179 |
| 1.19 | 0.091 | 0.175 |
| 1.29 | 0.083 | 0.145 |
| 1.26 | 0.108 | 0.154 |
| 1.32 | 0.091 | 0.145 |
| 1.32 | 0.087 | 0.145 |
| 1.40 | 0.091 | 0.150 |
| 1.38 | 0.087 | 0.150 |
| 1.40 | 0.108 | 0.158 |
| 1.44 | 0.120 | 0.125 |
| 1.35 | 0.112 | 0.133 |
| 1.33 | 0.920 | 0.157 |

7kg resistance

| step length | flight time | contact time |
|---|---|---|
| 1.00 | 0.037 | 0.225 |
| 1.21 | 0.083 | 0.183 |
| 1.17 | 0.058 | 0.137 |
| 1.42 | 0.083 | 0.162 |
| 1.23 | 0.075 | 0.145 |
| 1.51 | 0.087 | 0.137 |
| 1.34 | 0.083 | 0.137 |
| 1.54 | 0.087 | 0.137 |
| 1.55 | 0.100 | 0.129 |

14kg resistance

| step length | flight time | contact time |
|---|---|---|
| 0.91 | 0.054 | 0.216 |
| 1.24 | 0.079 | 0.158 |
| 1.16 | 0.066 | 0.141 |
| 1.41 | 0.079 | 0.150 |
| 1.26 | 0.083 | 0.141 |
| 1.51 | 0.070 | 0.129 |
| 1.48 | 0.100 | 0.150 |
| 1.57 | 0.095 | 0.120 |
| 1.42 | 0.112 | 0.116 |

| | | |
|---|---|---|
| 1.52 | 0.095 | 0.129 |
| 1.60 | 0.100 | 0.120 |
| 1.61 | 0.100 | 0.133 |
| 1.66 | 0.095 | 0.125 |

7kg resistance

| step length | flight time | contact time |
|---|---|---|
| 1.18 | 0.050 | 0.175 |
| 0.90 | 0.066 | 0.183 |
| 1.33 | 0.100 | 0.154 |
| 1.18 | 0.079 | 0.133 |
| 1.48 | 0.100 | 0.145 |
| 1.36 | 0.091 | 0.120 |
| 1.59 | 0.122 | 0.133 |
| 1.52 | 0.112 | 0.116 |
| 1.59 | 0.104 | 0.116 |
| 1.62 | 0.108 | 0.125 |
| 1.73 | 0.116 | 0.120 |
| 1.90 | 0.120 | 0.112 |
| 1.71 | 0.116 | 0.120 |
| 1.83 | 0.133 | 0.108 |

7kg resistance

| step length | flight time | contact time |
|---|---|---|
| 0.90 | 0.066 | 0.216 |
| 1.20 | 0.079 | 0.179 |
| 0.99 | 0.062 | 0.183 |
| 1.33 | 0.062 | 0.170 |
| 1.26 | 0.095 | 0.166 |
| 1.37 | 0.079 | 0.145 |
| 1.35 | 0.083 | 0.150 |
| 1.56 | 0.062 | 0.158 |
| 1.56 | 0.087 | 0.158 |
| 1.67 | 0.083 | 0.150 |
| 1.67 | 0.100 | 0.141 |
| 1.75 | 0.095 | 0.137 |
| 1.80 | 0.112 | 0.133 |
| 1.77 | 0.087 | 0.133 |

7kg resistance

| step length | flight time | contact time |
|---|---|---|
| 0.73 | 0.041 | 0.191 |
| 0.99 | 0.070 | 0.162 |
| 0.88 | 0.075 | 0.129 |
| 1.16 | 0.070 | 0.116 |

| | | |
|---|---|---|
| 1.55 | 0.100 | 0.120 |
| 1.52 | 0.100 | 0.125 |
| 1.53 | 0.108 | 0.120 |
| 1.56 | 0.095 | 0.116 |

14kg resistance

| step length | flight time | contact time |
|---|---|---|
| 1.18 | 0.050 | 0.175 |
| 0.81 | 0.062 | 0.208 |
| 1.27 | 0.083 | 0.162 |
| 1.01 | 0.079 | 0.150 |
| 1.34 | 0.083 | 0.150 |
| 1.18 | 0.087 | 0.158 |
| 1.14 | 0.108 | 0.125 |
| 1.26 | 0.095 | 0.137 |
| 1.44 | 0.091 | 0.150 |
| 1.31 | 0.095 | 0.141 |
| 1.46 | 0.104 | 0.137 |
| 1.40 | 0.104 | 0.133 |
| 1.45 | 0.108 | 0.129 |
| 1.52 | 0.104 | 0.133 |

14kg resistance

| step length | flight time | contact time |
|---|---|---|
| 0.92 | 0.045 | 0.212 |
| 1.34 | 0.070 | 0.191 |
| 1.08 | 0.083 | 0.154 |
| 1.43 | 0.070 | 0.141 |
| 1.32 | 0.075 | 0.158 |
| 1.54 | 0.079 | 0.145 |
| 1.47 | 0.100 | 0.141 |
| 1.54 | 0.087 | 0.125 |
| 1.54 | 0.108 | 0.125 |
| 1.55 | 0.104 | 0.120 |
| 1.59 | 0.108 | 0.120 |
| 1.53 | 0.108 | 0.116 |
| 1.66 | 0.112 | 0.116 |
| 1.48 | 0.100 | 0.120 |

14kg resistance

| step length | flight time | contact time |
|---|---|---|
| 0.53 | 0.062 | 0.170 |
| 0.85 | 0.075 | 0.145 |
| 0.71 | 0.058 | 0.133 |
| 1.07 | 0.066 | 0.162 |

| | | |
|---|---|---|
| 1.01 | 0.075 | 0.133 |
| 1.22 | 0.079 | 0.125 |
| 1.16 | 0.095 | 0.112 |
| 1.32 | 0.100 | 0.112 |
| 1.27 | 0.100 | 0.100 |
| 1.33 | 0.087 | 0.125 |
| 1.37 | 0.112 | 0.095 |
| 1.38 | 0.095 | 0.120 |
| 1.46 | 0.112 | 0.100 |
| 1.37 | 0.100 | 0.112 |

| | | |
|---|---|---|
| 0.87 | 0.070 | 0.150 |
| 1.17 | 0.066 | 0.137 |
| 0.93 | 0.079 | 0.145 |
| 1.29 | 0.087 | 0.129 |
| 1.12 | 0.091 | 0.133 |
| 1.37 | 0.087 | 0.129 |
| 1.30 | 0.075 | 0.129 |
| 1.42 | 0.095 | 0.137 |
| 1.40 | 0.087 | 0.129 |
| 1.47 | 0.091 | 0.129 |

| 7kg resistance | | |
|---|---|---|
| step length | flight time | contact time |
| 0.83 | 0.058 | 0.191 |
| 1.29 | 0.079 | 0.166 |
| 1.08 | 0.066 | 0.145 |
| 1.35 | 0.083 | 0.154 |
| 1.22 | 0.070 | 0.137 |
| 1.43 | 0.075 | 0.154 |
| 1.30 | 0.083 | 0.137 |
| 1.50 | 0.087 | 0.137 |
| 1.44 | 0.100 | 0.125 |
| 1.49 | 0.087 | 0.133 |
| 1.59 | 0.095 | 0.129 |
| 1.56 | 0.100 | 0.129 |
| 1.74 | 0.116 | 0.112 |
| 1.53 | 0.087 | 0.137 |

| 14kg resistance | | |
|---|---|---|
| step length | flight time | contact time |
| 0.76 | 0.033 | 0.179 |
| 1.18 | 0.075 | 0.175 |
| 0.94 | 0.079 | 0.150 |
| 1.38 | 0.083 | 0.133 |
| 1.19 | 0.087 | 0.137 |
| 1.41 | 0.079 | 0.137 |
| 1.35 | 0.095 | 0.137 |
| 1.43 | 0.083 | 0.125 |
| 1.41 | 0.100 | 0.129 |
| 1.38 | 0.083 | 0.120 |
| 1.42 | 0.100 | 0.120 |
| 1.36 | 0.091 | 0.116 |
| 1.46 | 0.083 | 0.116 |
| 1.38 | 0.091 | 0.129 |

| 7kg resistance | | |
|---|---|---|
| step length | flight time | contact time |
| 0.69 | 0.037 | 0.262 |
| 1.28 | 0.058 | 0.200 |
| 0.87 | 0.087 | 0.191 |
| 1.36 | 0.062 | 0.162 |
| 1.21 | 0.083 | 0.179 |
| 1.55 | 0.075 | 0.154 |
| 1.36 | 0.087 | 0.170 |
| 1.64 | 0.070 | 0.154 |
| 1.62 | 0.100 | 0.170 |
| 1.73 | 0.083 | 0.158 |
| 1.74 | 0.091 | 0.162 |
| 1.84 | 0.100 | 0.154 |
| 1.92 | 0.095 | 0.166 |
| 1.86 | 0.091 | 0.154 |

| 14kg resistance | | |
|---|---|---|
| step length | flight time | contact time |
| 0.90 | 0.070 | 0.237 |
| 1.35 | 0.100 | 0.183 |
| 1.10 | 0.091 | 0.162 |
| 1.39 | 0.075 | 0.154 |
| 1.25 | 0.100 | 0.162 |
| 1.46 | 0.087 | 0.133 |
| 1.36 | 0.104 | 0.158 |
| 1.53 | 0.100 | 0.137 |
| 1.52 | 0.108 | 0.150 |
| 1.54 | 0.095 | 0.133 |
| 1.64 | 0.116 | 0.145 |
| 1.50 | 0.108 | 0.129 |
| 1.66 | 0.108 | 0.141 |
| 1.50 | 0.100 | 0.129 |

| 7kg resistance | | |
|---|---|---|
| step length | flight time | contact time |
| 1.27 | 0.062 | 0.225 |
| 1.05 | 0.066 | 0.179 |
| 1.35 | 0.066 | 0.175 |
| 1.31 | 0.066 | 0.166 |
| 1.48 | 0.070 | 0.166 |
| 1.43 | 0.091 | 0.150 |
| 1.61 | 0.095 | 0.141 |
| 1.55 | 0.095 | 0.141 |
| 1.63 | 0.087 | 0.141 |
| 1.63 | 0.091 | 0.141 |
| 1.70 | 0.100 | 0.137 |
| 1.76 | 0.100 | 0.133 |
| 1.73 | 0.100 | 0.141 |
| 1.84 | 0.095 | 0.141 |

| 14kg resistance | | |
|---|---|---|
| step length | flight time | contact time |
| 1.27 | 0.025 | 0.220 |
| 0.92 | 0.045 | 0.216 |
| 1.31 | 0.050 | 0.195 |
| 1.15 | 0.058 | 0.187 |
| 1.37 | 0.050 | 0.183 |
| 1.21 | 0.079 | 0.179 |
| 1.44 | 0.070 | 0.154 |
| 1.32 | 0.075 | 0.162 |
| 1.42 | 0.054 | 0.166 |
| 1.45 | 0.083 | 0.166 |
| 1.47 | 0.070 | 0.166 |
| 1.44 | 0.079 | 0.166 |
| 1.43 | 0.075 | 0.154 |
| 1.55 | 0.070 | 0.154 |

| 7kg resistance | | |
|---|---|---|
| step length | flight time | contact time |
| 0.56 | 0.045 | 0.208 |
| 1.05 | 0.054 | 0.154 |
| 0.94 | 0.050 | 0.158 |
| 1.25 | 0.062 | 0.154 |
| 1.06 | 0.062 | 0.145 |
| 1.38 | 0.075 | 0.137 |
| 1.21 | 0.087 | 0.129 |
| 1.36 | 0.070 | 0.125 |
| 1.33 | 0.100 | 0.133 |
| 1.36 | 0.087 | 0.108 |
| 1.44 | 0.095 | 0.120 |
| 1.40 | 0.104 | 0.116 |
| 1.44 | 0.095 | 0.112 |
| 1.38 | 0.095 | 0.112 |

| 14kg resistance | | |
|---|---|---|
| step length | flight time | contact time |
| 0.98 | 0.050 | 0.183 |
| 0.70 | 0.070 | 0.170 |
| 1.16 | 0.050 | 0.145 |
| 0.81 | 0.066 | 0.166 |
| 1.10 | 0.091 | 0.137 |
| 0.67 | 0.066 | 0.141 |
| 1.59 | 0.079 | 0.137 |
| 1.05 | 0.083 | 0.137 |
| 1.23 | 0.095 | 0.120 |
| 1.15 | 0.087 | 0.129 |
| 1.29 | 0.087 | 0.129 |
| 1.17 | 0.100 | 0.133 |
| 1.33 | 0.087 | 0.116 |
| 1.33 | 0.104 | 0.120 |

| 7kg resistance | | |
|---|---|---|
| step length | flight time | contact time |
| 0.61 | 0.087 | 0.225 |
| 1.37 | 0.125 | 0.158 |
| 0.99 | 0.087 | 0.133 |
| 1.59 | 0.095 | 0.141 |
| 1.42 | 0.095 | 0.137 |
| 1.75 | 0.116 | 0.133 |
| 1.54 | 0.112 | 0.129 |
| 1.98 | 0.133 | 0.108 |
| 1.80 | 0.116 | 0.108 |

| 14kg resistance | | |
|---|---|---|
| step length | flight time | contact time |
| 1.36 | 0.062 | 0.229 |
| 1.20 | 0.133 | 0.193 |
| 1.38 | 0.066 | 0.129 |
| 1.37 | 0.116 | 0.158 |
| 1.56 | 0.108 | 0.120 |
| 1.52 | 0.120 | 0.133 |
| 1.67 | 0.100 | 0.129 |
| 1.61 | 0.145 | 0.125 |
| 1.57 | 0.104 | 0.112 |

| | | |
|---|---|---|
| 2.10 | 0.116 | 0.108 |
| 2.05 | 0.116 | 0.125 |
| 2.16 | 0.120 | 0.108 |
| 2.27 | 0.120 | 0.108 |
| 2.31 | 0.129 | 0.112 |

| | | |
|---|---|---|
| 1.75 | 0.145 | 0.116 |
| 1.54 | 0.104 | 0.112 |
| 1.73 | 0.133 | 0.112 |
| 1.59 | 0.116 | 0.108 |
| 1.89 | 0.137 | 0.108 |

7kg resistance

| step length | flight time | contact time |
|---|---|---|
| 0.64 | 0.029 | 0.229 |
| 1.23 | 0.062 | 0.175 |
| 1.01 | 0.062 | 0.170 |
| 1.33 | 0.066 | 0.170 |
| 1.24 | 0.075 | 0.150 |
| 1.46 | 0.083 | 0.150 |
| 1.43 | 0.075 | 0.145 |
| 1.52 | 0.087 | 0.141 |
| 1.57 | 0.087 | 0.133 |
| 1.56 | 0.091 | 0.141 |
| 1.59 | 0.095 | 0.120 |
| 1.64 | 0.112 | 0.120 |
| 1.68 | 0.104 | 0.116 |
| 1.71 | 0.112 | 0.125 |

14kg resistance

| step length | flight time | contact time |
|---|---|---|
| 0.77 | 0.037 | 0.195 |
| 1.33 | 0.062 | 0.187 |
| 1.06 | 0.062 | 0.170 |
| 1.44 | 0.083 | 0.166 |
| 1.20 | 0.079 | 0.145 |
| 1.50 | 0.075 | 0.154 |
| 1.40 | 0.079 | 0.154 |
| 1.47 | 0.095 | 0.137 |
| 1.50 | 0.108 | 0.129 |
| 1.51 | 0.104 | 0.125 |
| 1.52 | 0.100 | 0.125 |
| 1.55 | 0.104 | 0.129 |
| 1.64 | 0.104 | 0.120 |
| 1.55 | 0.112 | 0.125 |

7kg resistance

| step length | flight time | contact time |
|---|---|---|
| 1.28 | 0.079 | 0.195 |
| 0.98 | 0.070 | 0.170 |
| 1.29 | 0.079 | 0.166 |
| 1.29 | 0.075 | 0.150 |
| 1.49 | 0.079 | 0.154 |
| 1.44 | 0.079 | 0.150 |
| 1.56 | 0.079 | 0.141 |
| 1.54 | 0.083 | 0.145 |
| 1.71 | 0.087 | 0.141 |
| 1.74 | 0.091 | 0.133 |
| 1.79 | 0.095 | 0.145 |
| 1.80 | 0.091 | 0.129 |
| 1.86 | 0.100 | 0.137 |
| 1.91 | 0.087 | 0.133 |

14kg resistance

| step length | flight time | contact time |
|---|---|---|
| 1.04 | 0.062 | 0.187 |
| 1.19 | 0.062 | 0.179 |
| 1.20 | 0.087 | 0.162 |
| 1.30 | 0.079 | 0.141 |
| 1.34 | 0.095 | 0.145 |
| 1.38 | 0.091 | 0.137 |
| 1.42 | 0.091 | 0.137 |
| 1.48 | 0.087 | 0.141 |
| 1.47 | 0.083 | 0.145 |
| 1.56 | 0.100 | 0.137 |
| 1.58 | 0.083 | 0.145 |
| 1.53 | 0.095 | 0.150 |
| 1.65 | 0.087 | 0.141 |
| 1.58 | 0.116 | 0.133 |

7kg resistance

| step length | flight time | contact time |
|---|---|---|
| 1.23 | 0.079 | 0.166 |
| 1.12 | 0.112 | 0.175 |
| 1.37 | 0.100 | 0.116 |

14kg resistance

| step length | flight time | contact time |
|---|---|---|
| 1.19 | 0.091 | 0.179 |
| 1.02 | 0.100 | 0.170 |
| 1.25 | 0.079 | 0.162 |

| | | |
|---|---|---|
| 1.39 | 0.104 | 0.129 |
| 1.55 | 0.108 | 0.133 |
| 1.61 | 0.112 | 0.133 |
| 1.61 | 0.112 | 0.116 |
| 1.59 | 0.116 | 0.120 |
| 1.62 | 0.108 | 0.116 |
| 1.71 | 0.120 | 0.116 |
| 1.73 | 0.112 | 0.116 |
| 1.79 | 0.125 | 0.116 |
| 1.77 | 0.120 | 0.116 |
| 1.92 | 0.120 | 0.116 |

| | | |
|---|---|---|
| 1.19 | 0.104 | 0.166 |
| 1.34 | 0.100 | 0.145 |
| 1.31 | 0.112 | 0.141 |
| 1.41 | 0.079 | 0.150 |
| 1.36 | 0.095 | 0.166 |
| 1.44 | 0.087 | 0.154 |
| 1.45 | 0.100 | 0.154 |
| 1.42 | 0.095 | 0.150 |
| 1.54 | 0.120 | 0.145 |
| 1.43 | 0.100 | 0.141 |
| 1.56 | 0.108 | 0.154 |

| | 7kg assistance | | | 9kg assistance | | |
|---|---|---|---|---|---|---|
| [illegible] | step length | flight time | contact time | step length | flight time | contact time |
| | 1.02 | 0.078 | 0.200 | 1.34 | 0.116 | 0.141 |
| | 1.33 | 0.112 | 0.141 | 1.54 | 0.112 | 0.120 |
| | 1.40 | 0.116 | 0.120 | 1.57 | 0.125 | 0.116 |
| | 1.60 | 0.108 | 0.133 | 1.83 | 0.129 | 0.104 |
| | 1.67 | 0.125 | 0.116 | 1.82 | 0.141 | 0.091 |
| | 1.81 | 0.137 | 0.104 | 1.93 | 0.129 | 0.104 |
| | 1.78 | 0.125 | 0.100 | 1.93 | 0.125 | 0.091 |
| | 1.94 | 0.129 | 0.095 | 2.07 | 0.133 | 0.095 |
| | 1.93 | 0.133 | 0.091 | 2.10 | 0.125 | 0.091 |
| | 2.11 | 0.137 | 0.091 | 2.21 | 0.125 | 0.087 |
| | 2.13 | 0.125 | 0.083 | 2.37 | 0.133 | 0.087 |
| | 2.19 | 0.129 | 0.100 | 2.59 | 0.137 | 0.083 |
| | 2.40 | 0.137 | 0.075 | 2.56 | 0.133 | 0.087 |
| | 2.43 | 0.137 | 0.095 | 2.67 | 0.137 | 0.087 |

| | 7kg assistance | | | 9kg assistance | | |
|---|---|---|---|---|---|---|
| [illegible] | step length | flight time | contact time | step length | flight time | contact time |
| | 1.31 | 0.062 | 0.175 | 1.31 | 0.070 | 0.183 |
| | 1.22 | 0.100 | 0.154 | 1.23 | 0.083 | 0.141 |
| | 1.49 | 0.104 | 0.116 | 1.53 | 0.087 | 0.133 |
| | 1.43 | 0.091 | 0.116 | 1.51 | 0.087 | 0.125 |
| | 1.66 | 0.095 | 0.125 | 1.69 | 0.104 | 0.129 |
| | 1.70 | 0.116 | 0.116 | 1.65 | 0.095 | 0.108 |
| | 1.70 | 0.112 | 0.104 | 1.82 | 0.095 | 0.116 |
| | 1.75 | 0.100 | 0.104 | 1.86 | 0.104 | 0.116 |
| | 1.82 | 0.108 | 0.108 | 1.88 | 0.104 | 0.116 |
| | 1.83 | 0.100 | 0.116 | 1.94 | 0.104 | 0.112 |
| | 1.89 | 0.108 | 0.116 | 1.88 | 0.108 | 0.108 |
| | 1.95 | 0.112 | 0.108 | 1.97 | 0.112 | 0.108 |
| | 1.98 | 0.129 | 0.104 | 1.90 | 0.120 | 0.095 |
| | 1.98 | 0.116 | 0.095 | 2.12 | 0.120 | 0.095 |

| | 7kg assistance | | | 9kg assistance | | |
|---|---|---|---|---|---|---|
| [illegible] | step length | flight time | contact time | step length | flight time | contact time |
| | 1.36 | 0.087 | 0.179 | 1.26 | 0.075 | 0.158 |
| | 1.30 | 0.095 | 0.158 | 1.36 | 0.104 | 0.154 |
| | 1.56 | 0.120 | 0.125 | 1.56 | 0.120 | 0.120 |
| | 1.49 | 0.112 | 0.125 | 1.47 | 0.095 | 0.120 |
| | 1.64 | 0.112 | 0.116 | 1.62 | 0.108 | 0.112 |
| | 1.66 | 0.112 | 0.116 | 1.58 | 0.095 | 0.112 |
| | 1.76 | 0.112 | 0.104 | 1.72 | 0.100 | 0.108 |
| | 1.75 | 0.116 | 0.108 | 1.81 | 0.112 | 0.112 |
| | 1.82 | 0.133 | 0.095 | 1.81 | 0.116 | 0.104 |

| | | | | | |
|---|---|---|---|---|---|
| 1.88 | 0.125 | 0.095 | 1.83 | 0.112 | 0.095 |
| 1.88 | 0.133 | 0.100 | 1.82 | 0.116 | 0.104 |
| 1.93 | 0.129 | 0.091 | 1.92 | 0.112 | 0.095 |
| 1.85 | 0.129 | 0.100 | 1.89 | 0.129 | 0.091 |

| 7kg assistance | | | 9kg assistance | | |
|---|---|---|---|---|---|
| step length | flight time | contact time | step length | flight time | contact time |
| 1.03 | 0.075 | 0.187 | 0.98 | 0.087 | 0.204 |
| 1.33 | 0.104 | 0.175 | 1.41 | 0.116 | 0.154 |
| 1.35 | 0.125 | 0.112 | 1.31 | 0.108 | 0.150 |
| 1.53 | 0.120 | 0.137 | 1.71 | 0.145 | 0.133 |
| 1.53 | 0.116 | 0.116 | 1.60 | 0.112 | 0.120 |
| 1.89 | 0.141 | 0.116 | 1.91 | 0.154 | 0.120 |
| 1.76 | 0.125 | 0.116 | 1.83 | 0.129 | 0.104 |
| 2.08 | 0.154 | 0.112 | 2.16 | 0.145 | 0.120 |
| 1.96 | 0.137 | 0.095 | 2.21 | 0.150 | 0.108 |
| 2.23 | 0.158 | 0.095 | 2.27 | 0.145 | 0.108 |
| 2.32 | 0.150 | 0.095 | 2.39 | 0.158 | 0.095 |
| 2.54 | 0.175 | 0.095 | 2.50 | 0.154 | 0.104 |
| 2.61 | 0.154 | 0.100 | 2.64 | 0.158 | 0.100 |
| 2.62 | 0.158 | 0.100 | 2.56 | 0.158 | 0.095 |

| 7kg assistance | | | 9kg assistance | | |
|---|---|---|---|---|---|
| step length | flight time | contact time | step length | flight time | contact time |
| 1.24 | 0.070 | 0.237 | 1.37 | 0.075 | 0.175 |
| 1.25 | 0.058 | 0.183 | 1.29 | 0.066 | 0.170 |
| 1.54 | 0.083 | 0.170 | 1.62 | 0.087 | 0.154 |
| 1.49 | 0.087 | 0.145 | 1.54 | 0.091 | 0.133 |
| 1.70 | 0.100 | 0.133 | 1.72 | 0.087 | 0.125 |
| 1.69 | 0.104 | 0.120 | 1.69 | 0.095 | 0.125 |
| 1.83 | 0.112 | 0.120 | 1.81 | 0.100 | 0.120 |
| 1.91 | 0.116 | 0.108 | 1.87 | 0.108 | 0.112 |
| 1.92 | 0.125 | 0.112 | 1.89 | 0.108 | 0.112 |
| 1.93 | 0.120 | 0.104 | 1.94 | 0.112 | 0.108 |
| 1.93 | 0.125 | 0.108 | 1.86 | 0.108 | 0.104 |
| 1.95 | 0.116 | 0.108 | 1.94 | 0.112 | 0.104 |
| 1.85 | 0.112 | 0.112 | 1.91 | 0.112 | 0.104 |
| 2.05 | 0.116 | 0.112 | 2.04 | 0.112 | 0.104 |

| 7kg assistance | | | 9kg assistance | | |
|---|---|---|---|---|---|
| step length | flight time | contact time | step length | flight time | contact time |
| 1.22 | 0.058 | 0.195 | 1.24 | 0.050 | 0.166 |
| 1.00 | 0.050 | 0.170 | 1.07 | 0.054 | 0.175 |
| 1.48 | 0.079 | 0.154 | 1.51 | 0.083 | 0.141 |
| 1.25 | 0.087 | 0.137 | 1.33 | 0.079 | 0.133 |

| | | |
|---|---|---|
| 1.59 | 0.091 | 0.120 |
| 1.52 | 0.095 | 0.125 |
| 1.71 | 0.095 | 0.120 |
| 1.76 | 0.108 | 0.125 |
| 1.87 | 0.112 | 0.120 |
| 1.96 | 0.133 | 0.120 |
| 1.82 | 0.116 | 0.104 |
| 2.01 | 0.129 | 0.108 |
| 1.71 | 0.125 | 0.112 |
| 2.08 | 0.129 | 0.108 |

| | | |
|---|---|---|
| 1.65 | 0.079 | 0.137 |
| 1.63 | 0.100 | 0.125 |
| 1.77 | 0.095 | 0.120 |
| 1.78 | 0.108 | 0.125 |
| 1.87 | 0.116 | 0.104 |
| 1.94 | 0.120 | 0.108 |
| 1.84 | 0.116 | 0.100 |
| 1.98 | 0.112 | 0.104 |
| 1.92 | 0.120 | 0.100 |
| 2.06 | 0.120 | 0.104 |

| | 7kg assistance | |
|---|---|---|
| step length | flight time | contact time |
| 1.14 | 0.058 | 0.150 |
| 1.11 | 0.079 | 0.150 |
| 1.48 | 0.091 | 0.125 |
| 1.31 | 0.087 | 0.120 |
| 1.59 | 0.095 | 0.116 |
| 1.56 | 0.108 | 0.104 |
| 1.65 | 0.112 | 0.104 |
| 1.64 | 0.108 | 0.087 |
| 1.75 | 0.108 | 0.095 |
| 1.77 | 0.120 | 0.095 |
| 1.71 | 0.108 | 0.083 |
| 1.82 | 0.108 | 0.091 |
| 1.81 | 0.116 | 0.095 |
| 1.86 | 0.100 | 0.091 |

| | 9kg assistance | |
|---|---|---|
| step length | flight time | contact time |
| 1.04 | 0.095 | 0.191 |
| 1.07 | 0.083 | 0.137 |
| 1.40 | 0.104 | 0.141 |
| 1.35 | 0.083 | 0.125 |
| 1.49 | 0.091 | 0.129 |
| 1.55 | 0.095 | 0.116 |
| 1.64 | 0.108 | 0.104 |
| 1.66 | 0.108 | 0.095 |
| 1.67 | 0.104 | 0.095 |
| 1.76 | 0.112 | 0.091 |
| 1.74 | 0.120 | 0.091 |
| 1.85 | 0.108 | 0.083 |
| 1.77 | 0.116 | 0.087 |
| 1.95 | 0.116 | 0.083 |

| | 7kg assistance | |
|---|---|---|
| step length | flight time | contact time |
| 1.39 | 0.075 | 0.220 |
| 1.30 | 0.079 | 0.175 |
| 1.53 | 0.087 | 0.158 |
| 1.52 | 0.091 | 0.141 |
| 1.68 | 0.108 | 0.141 |
| 1.64 | 0.100 | 0.125 |
| 1.66 | 0.100 | 0.133 |
| 1.67 | 0.091 | 0.120 |
| 1.76 | 0.108 | 0.125 |
| 1.76 | 0.095 | 0.116 |
| 1.83 | 0.112 | 0.125 |
| 1.84 | 0.104 | 0.112 |
| 1.82 | 0.112 | 0.120 |
| 1.83 | 0.091 | 0.112 |

| | 9kg assistance | |
|---|---|---|
| step length | flight time | contact time |
| 1.42 | 0.083 | 0.208 |
| 1.30 | 0.083 | 0.179 |
| 1.60 | 0.112 | 0.154 |
| 1.39 | 0.079 | 0.141 |
| 1.67 | 0.095 | 0.150 |
| 1.62 | 0.087 | 0.125 |
| 1.65 | 0.104 | 0.137 |
| 1.61 | 0.087 | 0.116 |
| 1.72 | 0.108 | 0.120 |
| 1.70 | 0.091 | 0.112 |
| 1.78 | 0.104 | 0.120 |
| 1.77 | 0.091 | 0.112 |
| 1.80 | 0.104 | 0.120 |
| 1.90 | 0.100 | 0.116 |

| 7kg assistance | | | 9kg assistance | | |
|---|---|---|---|---|---|
| step length | flight time | contact time | step length | flight time | contact time |
| 0.99 | 0.050 | 0.158 | 0.89 | 0.041 | 0.191 |
| 1.51 | 0.075 | 0.183 | 1.50 | 0.075 | 0.183 |
| 1.20 | 0.079 | 0.154 | 1.30 | 0.083 | 0.158 |
| 1.54 | 0.087 | 0.145 | 1.68 | 0.095 | 0.154 |
| 1.41 | 0.087 | 0.129 | 1.44 | 0.087 | 0.129 |
| 1.81 | 0.100 | 0.133 | 1.91 | 0.112 | 0.137 |
| 1.73 | 0.112 | 0.120 | 1.72 | 0.100 | 0.125 |
| 1.97 | 0.112 | 0.125 | 2.02 | 0.116 | 0.120 |
| 1.90 | 0.100 | 0.120 | 1.91 | 0.100 | 0.116 |
| 2.13 | 0.125 | 0.116 | 2.20 | 0.112 | 0.125 |
| 2.06 | 0.108 | 0.108 | 2.24 | 0.112 | 0.125 |
| 2.32 | 0.125 | 0.112 | 2.27 | 0.112 | 0.116 |
| 2.43 | 0.125 | 0.112 | 2.49 | 0.116 | 0.112 |
| 2.27 | 0.120 | 0.100 | 2.54 | 0.133 | 0.112 |

| 7kg assistance | | | 9kg assistance | | |
|---|---|---|---|---|---|
| step length | flight time | contact time | step length | flight time | contact time |
| 0.85 | 0.058 | 0.191 | 0.93 | 0.054 | 0.216 |
| 1.26 | 0.083 | 0.158 | 1.48 | 0.104 | 0.166 |
| 0.97 | 0.095 | 0.141 | 1.28 | 0.104 | 0.133 |
| 1.71 | 0.091 | 0.125 | 1.66 | 0.104 | 0.133 |
| 1.37 | 0.091 | 0.133 | 1.51 | 0.104 | 0.120 |
| 1.69 | 0.116 | 0.116 | 1.84 | 0.112 | 0.120 |
| 1.66 | 0.104 | 0.108 | 1.81 | 0.116 | 0.116 |
| 1.80 | 0.104 | 0.108 | 1.91 | 0.120 | 0.116 |
| 1.81 | 0.133 | 0.095 | 1.95 | 0.112 | 0.104 |
| 1.97 | 0.125 | 0.100 | 2.07 | 0.120 | 0.104 |
| 1.99 | 0.120 | 0.095 | 2.09 | 0.116 | 0.104 |
| 2.05 | 0.125 | 0.095 | 2.27 | 0.133 | 0.091 |
| 2.13 | 0.120 | 0.095 | 2.39 | 0.129 | 0.095 |
| 2.25 | 0.125 | 0.095 | 2.44 | 0.133 | 0.091 |

| 7kg assistance | | | 9kg assistance | | |
|---|---|---|---|---|---|
| step length | flight time | contact time | step length | flight time | contact time |
| 1.40 | 0.075 | 0.187 | 1.37 | 0.079 | 0.191 |
| 1.16 | 0.087 | 0.145 | 1.33 | 0.120 | 0.154 |
| 1.60 | 0.079 | 0.137 | 1.69 | 0.104 | 0.141 |
| 1.60 | 0.104 | 0.137 | 1.67 | 0.108 | 0.129 |
| 1.90 | 0.100 | 0.125 | 2.04 | 0.112 | 0.129 |
| 1.83 | 0.120 | 0.116 | 1.93 | 0.120 | 0.129 |
| 2.04 | 0.116 | 0.095 | 2.09 | 0.112 | 0.108 |
| 2.02 | 0.120 | 0.104 | 2.04 | 0.125 | 0.100 |
| 2.17 | 0.112 | 0.108 | 2.27 | 0.129 | 0.100 |

| | | | | | |
|---|---|---|---|---|---|
| 2.29 | 0.129 | 0.100 | 2.31 | 0.125 | 0.100 |
| 2.31 | 0.116 | 0.100 | 2.49 | 0.133 | 0.095 |
| 2.62 | 0.133 | 0.100 | 2.65 | 0.129 | 0.100 |
| 2.46 | 0.116 | 0.091 | 2.84 | 0.133 | 0.100 |
| 2.79 | 0.129 | 0.100 | 2.97 | 0.141 | 0.104 |

| 7kg assistance | | | 9kg assistance | | |
|---|---|---|---|---|---|
| step length | flight time | contact time | step length | flight time | contact time |
| 1.35 | 0.062 | 0.195 | 1.35 | 0.050 | 0.179 |
| 1.50 | 0.112 | 0.187 | 1.19 | 0.083 | 0.175 |
| 1.78 | 0.116 | 0.145 | 1.53 | 0.095 | 0.137 |
| 1.68 | 0.125 | 0.141 | 1.42 | 0.091 | 0.137 |
| 1.78 | 0.108 | 0.133 | 1.69 | 0.100 | 0.125 |
| 1.83 | 0.120 | 0.125 | 1.70 | 0.104 | 0.120 |
| 1.95 | 0.120 | 0.125 | 1.78 | 0.112 | 0.112 |
| 1.98 | 0.133 | 0.116 | 1.76 | 0.112 | 0.108 |
| 2.01 | 0.137 | 0.108 | 1.87 | 0.108 | 0.112 |
| 2.05 | 0.137 | 0.112 | 1.86 | 0.116 | 0.108 |
| 1.93 | 0.129 | 0.104 | 1.90 | 0.112 | 0.108 |
| 2.16 | 0.137 | 0.112 | 2.01 | 0.120 | 0.108 |
| 2.13 | 0.145 | 0.104 | 1.94 | 0.120 | 0.108 |
| 2.23 | 0.150 | 0.112 | 2.03 | 0.129 | 0.100 |

| 7kg assistance | | | 9kg assistance | | |
|---|---|---|---|---|---|
| step length | flight time | contact time | step length | flight time | contact time |
| 1.37 | 0.075 | 0.216 | 1.13 | 0.091 | 0.166 |
| 1.31 | 0.091 | 0.170 | 1.35 | 0.091 | 0.179 |
| 1.64 | 0.108 | 0.162 | 1.57 | 0.120 | 0.166 |
| 1.74 | 0.104 | 0.145 | 1.78 | 0.120 | 0.145 |
| 1.84 | 0.104 | 0.145 | 1.83 | 0.133 | 0.133 |
| 1.95 | 0.108 | 0.141 | 2.04 | 0.125 | 0.145 |
| 2.14 | 0.125 | 0.133 | 2.22 | 0.125 | 0.133 |
| 2.24 | 0.137 | 0.120 | 2.39 | 0.154 | 0.129 |
| 2.48 | 0.154 | 0.120 | 2.34 | 0.141 | 0.125 |
| 2.46 | 0.133 | 0.125 | 2.68 | 0.158 | 0.112 |
| 2.63 | 0.158 | 0.120 | 2.61 | 0.129 | 0.120 |
| 2.53 | 0.112 | 0.125 | 2.81 | 0.166 | 0.112 |
| 2.63 | 0.145 | 0.125 | 3.00 | 0.158 | 0.112 |
| 2.83 | 0.159 | 0.129 | 3.05 | 0.175 | 0.112 |

| 7kg assistance | | | 9kg assistance | | |
|---|---|---|---|---|---|
| step length | flight time | contact time | step length | flight time | contact time |
| 1.17 | 0.087 | 0.158 | 1.18 | 0.087 | 0.158 |
| 1.42 | 0.116 | 0.145 | 1.56 | 0.112 | 0.158 |
| 1.40 | 0.095 | 0.141 | 1.43 | 0.095 | 0.133 |

| | | | | | |
|---|---|---|---|---|---|
| 1.73 | 0.128 | 0.125 | 1.82 | 0.141 | 0.125 |
| 1.61 | 0.120 | 0.104 | 1.71 | 0.108 | 0.116 |
| 1.97 | 0.129 | 0.112 | 2.06 | 0.129 | 0.125 |
| 1.82 | 0.116 | 0.112 | 1.95 | 0.112 | 0.112 |
| 2.17 | 0.133 | 0.104 | 2.31 | 0.141 | 0.104 |
| 2.11 | 0.129 | 0.100 | 2.20 | 0.112 | 0.112 |
| 2.28 | 0.133 | 0.104 | 2.63 | 0.137 | 0.116 |
| 2.38 | 0.129 | 0.095 | 2.35 | 0.121 | 0.108 |
| 2.57 | 0.141 | 0.104 | 2.65 | 0.133 | 0.104 |
| 2.46 | 0.129 | 0.095 | 2.55 | 0.112 | 0.104 |
| 2.89 | 0.166 | 0.100 | 2.78 | 0.137 | 0.104 |

11kg assistance

| [illegible] | step length | flight time | contact time |
|---|---|---|---|
| | 0.98 | 0.070 | 0.191 |
| | 1.38 | 0.079 | 0.162 |
| | 1.38 | 0.104 | 0.141 |
| | 1.76 | 0.125 | 0.120 |
| | 1.72 | 0.129 | 0.112 |
| | 1.88 | 0.133 | 0.108 |
| | 1.89 | 0.125 | 0.091 |
| | 2.05 | 0.120 | 0.108 |
| | 1.97 | 0.137 | 0.091 |
| | 2.20 | 0.129 | 0.091 |
| | 2.21 | 0.129 | 0.091 |
| | 2.28 | 0.141 | 0.087 |
| | 2.39 | 0.129 | 0.083 |
| | 2.52 | 0.137 | 0.083 |

11kg assistance

| [illegible] | step length | flight time | contact time |
|---|---|---|---|
| | 1.29 | 0.083 | 0.179 |
| | 1.25 | 0.079 | 0.137 |
| | 1.54 | 0.091 | 0.129 |
| | 1.56 | 0.091 | 0.116 |
| | 1.78 | 0.104 | 0.125 |
| | 1.77 | 0.104 | 0.116 |
| | 1.88 | 0.108 | 0.112 |
| | 1.88 | 0.108 | 0.108 |
| | 1.90 | 0.108 | 0.104 |
| | 1.93 | 0.104 | 0.104 |
| | 2.00 | 0.120 | 0.104 |
| | 1.96 | 0.104 | 0.104 |
| | 1.97 | 0.112 | 0.104 |
| | 2.10 | 0.125 | 0.095 |

11kg assistance

| [illegible] | step length | flight time | contact time |
|---|---|---|---|
| | 1.22 | 0.079 | 0.189 |
| | 1.35 | 0.086 | 0.159 |
| | 1.50 | 0.094 | 0.145 |
| | 1.62 | 0.107 | 0.135 |
| | 1.74 | 0.110 | 0.123 |
| | 1.87 | 0.117 | 0.118 |
| | 1.89 | 0.114 | 0.114 |
| | 2.03 | 0.119 | 0.112 |
| | 2.07 | 0.124 | 0.109 |

| | | | |
|---|---|---|---|
| | 2.15 | 0.124 | 0.104 |
| | 2.24 | 0.126 | 0.103 |
| | 2.32 | 0.130 | 0.103 |
| | 2.38 | 0.130 | 0.101 |

11kg assistance

| [illegible] | step length | flight time | contact time |
|---|---|---|---|
| | 1.03 | 0.066 | 0.212 |
| | 1.37 | 0.116 | 0.158 |
| | 1.39 | 0.112 | 0.141 |
| | 1.62 | 0.129 | 0.129 |
| | 1.67 | 0.125 | 0.120 |
| | 1.88 | 0.141 | 0.116 |
| | 1.91 | 0.129 | 0.108 |
| | 2.08 | 0.137 | 0.112 |
| | 2.23 | 0.158 | 0.104 |
| | 2.19 | 0.137 | 0.108 |
| | 2.43 | 0.145 | 0.108 |
| | 2.30 | 0.141 | 0.091 |
| | 2.72 | 0.154 | 0.104 |
| | 2.60 | 0.150 | 0.100 |

11kg assistance

| [illegible] | step length | flight time | contact time |
|---|---|---|---|
| | 1.31 | 0.075 | 0.162 |
| | 1.30 | 0.066 | 0.158 |
| | 1.57 | 0.079 | 0.158 |
| | 1.57 | 0.087 | 0.137 |
| | 1.74 | 0.095 | 0.133 |
| | 1.75 | 0.104 | 0.116 |
| | 1.82 | 0.108 | 0.116 |
| | 1.84 | 0.108 | 0.104 |
| | 1.93 | 0.116 | 0.112 |
| | 1.94 | 0.108 | 0.104 |
| | 1.86 | 0.095 | 0.108 |
| | 1.99 | 0.100 | 0.116 |
| | 2.01 | 0.116 | 0.112 |
| | 2.00 | 0.100 | 0.104 |

11kg assistance

| [illegible] | step length | flight time | contact time |
|---|---|---|---|
| | 1.25 | 0.058 | 0.158 |
| | 1.07 | 0.045 | 0.170 |
| | 1.56 | 0.079 | 0.158 |
| | 1.46 | 0.095 | 0.137 |

| | | |
|---|---|---|
| 1.59 | 0.083 | 0.125 |
| 1.71 | 0.104 | 0.120 |
| 1.80 | 0.095 | 0.125 |
| 1.83 | 0.104 | 0.120 |
| 1.99 | 0.125 | 0.116 |
| 1.96 | 0.125 | 0.104 |
| 1.97 | 0.129 | 0.100 |
| 2.09 | 0.125 | 0.104 |
| 2.00 | 0.133 | 0.100 |
| 2.10 | 0.120 | 0.100 |

| | 11kg assistance | |
|---|---|---|
| step length | flight time | contact time |
| 1.22 | 0.079 | 0.189 |
| 1.35 | 0.086 | 0.159 |
| 1.50 | 0.094 | 0.145 |
| 1.62 | 0.107 | 0.135 |
| 1.74 | 0.110 | 0.123 |
| 1.87 | 0.117 | 0.118 |
| 1.89 | 0.114 | 0.114 |
| 2.03 | 0.119 | 0.112 |
| 2.07 | 0.124 | 0.109 |
| 2.15 | 0.124 | 0.104 |
| 2.24 | 0.126 | 0.103 |
| 2.32 | 0.130 | 0.103 |
| 2.38 | 0.130 | 0.101 |

| | 11kg assistance | |
|---|---|---|
| step length | flight time | contact time |
| 1.50 | 0.083 | 0.200 |
| 1.37 | 0.079 | 0.162 |
| 1.76 | 0.120 | 0.154 |
| 1.60 | 0.091 | 0.137 |
| 1.87 | 0.112 | 0.137 |
| 1.87 | 0.112 | 0.129 |
| 1.90 | 0.112 | 0.129 |
| 1.87 | 0.100 | 0.120 |
| 2.00 | 0.116 | 0.125 |
| 1.97 | 0.112 | 0.116 |
| 1.93 | 0.112 | 0.116 |
| 2.09 | 0.120 | 0.112 |
| 1.93 | 0.108 | 0.112 |
| 2.07 | 0.116 | 0.108 |

11kg assistance

| [illegible] | step length | flight time | contact time |
|---|---|---|---|
| | 0.96 | 0.062 | 0.200 |
| | 1.46 | 0.079 | 0.166 |
| | 1.34 | 0.083 | 0.158 |
| | 1.68 | 0.100 | 0.133 |
| | 1.58 | 0.095 | 0.129 |
| | 1.97 | 0.116 | 0.120 |
| | 1.82 | 0.108 | 0.116 |
| | 2.09 | 0.116 | 0.116 |
| | 2.14 | 0.108 | 0.116 |
| | 2.21 | 0.125 | 0.108 |
| | 2.49 | 0.133 | 0.100 |
| | 2.55 | 0.141 | 0.104 |
| | 2.65 | 0.129 | 0.095 |
| | 2.69 | 0.125 | 0.112 |

11kg assistance

| [illegible] | step length | flight time | contact time |
|---|---|---|---|
| | 0.93 | 0.075 | 0.216 |
| | 1.42 | 0.095 | 0.154 |
| | 1.18 | 0.087 | 0.133 |
| | 1.61 | 0.104 | 0.129 |
| | 1.50 | 0.100 | 0.129 |
| | 1.81 | 0.095 | 0.112 |
| | 1.75 | 0.116 | 0.125 |
| | 2.00 | 0.116 | 0.100 |
| | 1.93 | 0.120 | 0.108 |
| | 2.03 | 0.104 | 0.100 |
| | 2.10 | 0.116 | 0.100 |
| | 2.19 | 0.116 | 0.100 |
| | 2.35 | 0.120 | 0.100 |
| | 2.40 | 0.108 | 0.100 |

11kg assistance

| [illegible] | step length | flight time | contact time |
|---|---|---|---|
| | 1.52 | 0.116 | 0.166 |
| | 1.21 | 0.091 | 0.150 |
| | 1.80 | 0.108 | 0.141 |
| | 1.64 | 0.120 | 0.129 |
| | 2.00 | 0.112 | 0.112 |
| | 2.02 | 0.133 | 0.116 |
| | 2.20 | 0.120 | 0.112 |
| | 2.29 | 0.133 | 0.108 |
| | 2.29 | 0.125 | 0.104 |

| | | |
|---|---|---|
| 2.40 | 0.133 | 0.104 |
| 2.81 | 0.137 | 0.100 |
| 3.02 | 0.154 | 0.100 |
| 3.05 | 0.154 | 0.095 |
| 3.10 | 0.129 | 0.108 |

11kg assistance

| step length | flight time | contact time |
|---|---|---|
| 1.41 | 0.066 | 0.204 |
| 1.22 | 0.083 | 0.170 |
| 1.56 | 0.075 | 0.133 |
| 1.50 | 0.104 | 0.145 |
| 1.76 | 0.104 | 0.112 |
| 1.75 | 0.112 | 0.116 |
| 1.90 | 0.112 | 0.108 |
| 1.88 | 0.116 | 0.108 |
| 1.89 | 0.112 | 0.104 |
| 1.99 | 0.125 | 0.104 |
| 2.00 | 0.125 | 0.100 |
| 2.11 | 0.133 | 0.100 |
| 2.06 | 0.133 | 0.100 |
| 2.09 | 0.120 | 0.100 |

11kg assistance

| step length | flight time | contact time |
|---|---|---|
| 1.25 | 0.095 | 0.225 |
| 1.52 | 0.112 | 0.175 |
| 1.49 | 0.095 | 0.162 |
| 1.73 | 0.112 | 0.166 |
| 1.93 | 0.128 | 0.137 |
| 2.05 | 0.120 | 0.133 |
| 1.94 | 0.125 | 0.120 |
| 2.27 | 0.129 | 0.129 |
| 2.36 | 0.145 | 0.116 |
| 2.57 | 0.158 | 0.100 |
| 2.65 | 0.154 | 0.104 |
| 2.69 | 0.150 | 0.112 |
| 2.84 | 0.141 | 0.112 |
| 3.04 | 0.166 | 0.104 |

11kg assistance

| step length | flight time | contact time |
|---|---|---|
| 1.16 | 0.095 | 0.150 |
| 1.58 | 0.108 | 0.150 |
| 1.48 | 0.100 | 0.129 |

| | | |
|---|---|---|
| 1.76 | 0.129 | 0.137 |
| 1.74 | 0.129 | 0.108 |
| 1.96 | 0.133 | 0.116 |
| 1.88 | 0.108 | 0.108 |
| 2.22 | 0.141 | 0.108 |
| 2.23 | 0.116 | 0.104 |
| 2.35 | 0.133 | 0.108 |
| 2.39 | 0.112 | 0.108 |
| 2.53 | 0.137 | 0.100 |
| 2.55 | 0.129 | 0.100 |
| 2.78 | 0.129 | 0.100 |
